# Supplementary material for: Long-range charge transfer mechanism of the III2IV2 mycobacterial supercomplex
Source: Nat Commun. 2024 Jun 20;15:5276. doi: 10.1038/s41467-024-49628-9 (PMC11189923; doi:10.1038/s41467-024-49628-9)
Supplement: Supplementary file 1 — Supplementary Information [file 41467_2024_49628_MOESM1_ESM.pdf]

Supplementary Information

for

Long-Range Charge Transfer Mechanism of the III_2IV_2 Mycobacterial Supercomplex

Daniel Riepl[1,a], Ana P. Gamiz-Hernandez[1,a], Terezia Kovalova[1,a], Sylwia M. Król, Sophie L. Mader[1], Dan Sjöstrand[1], Martin Högbom[1], Peter Brzezinski[1], Ville R. I. Kaila[1,*]

Content

Extended discussion: Functional relevance of mycobacterial adaptions at the Qo site

Figure S1. Details of the structural model of the SC.
Figure S2. Molecular dynamics setup of the *Mycobacterium smegmatis* III_2IV_2 supercomplex.
Figure S3. B-factors calculated from MD simulations and from cryoEM experiments.
Figure S4. SodC interaction and dynamics.
Figure S5. Dynamics of buried water molecules in the SC.
Figure S6. Comparison of MD and cryoEM water molecules**.**
Figure S7. Example cryoEM densities and BNC models based on the 2.3 Å map.
Figure S8. Quinone dynamics in the Q_o sites.
Figure S9. Dynamics of the PDFY loop in the Q_{o1a} site.
Figure S10. Example cryoEM densities of key regions based on the of the 2.8 Å map model.
Figure S11. QM/MM free energy simulations of the PCET reactions in the Q_{o1a} site.
Figure S12. Details of the electron transfer rate calculations.
Figure S13. Kinetic models of electron transfer in the supercomplex.
Figure S14. QM/MM dynamics at the Q_i site.
Figure S15. CryoEM data analysis and validation of the 2.3 Å map.
Figure S16. CryoEM data analysis and validation of the 2.8 Å map.
Figure S17. Example cryoEM densities of lipid modelled based on the of the 2.3 Å map model.
Figure S18. Hydration dynamics in the D- and K-channel.
Figure S19. Computed redox potentials around the Q_{o1a} site.
Figure S20. Multiple sequence alignment of QcrB.
Figure S21. Forcefield benchmarking and parameterisation.
Figure S22. SC activity.
Figure S23. Force field validation.
Figure S24. Schematic overview of charge transfer and quinone diffusion energetics.

Table S1. List of atomistic molecular dynamics simulations (*ca.* 10 μs).
Table S2. List of subunits in the simulation models.
Table S3. List of residues modelled in non-standard protonation states based on Monte Carlo electrostatic calculations.
Table S4. Calculated electron transfer rates and estimated parameters.
Table S5. Calculated average redox midpoint potentials of cofactors based on MD simulations.
Table S6. Cryo-EM data collection, refinement, and validation statistics.
Table S7. Atom types and partial charges for MQ cofactors.
Table S8. Atom types and partial charges for the FeS centre.
Table S9. Atom types and partial charges for heme *b*.
Table S10. Atom types and partial charges for heme *c*.
Table S11. Atom types and partial charges for Cu active site in SodC.
Table S12. Validation of the menaquinone geometry.
Table S13. Validation of the FeS geometry.
Table S14. Validation of heme *b* geometry.
Table S15. Calculated pK_a values of selected residues in the Q_{o1a} and Q_i sites.

Movie S1. Molecular dynamics of the SC from *M. smegmatis.*
Movie S2. Quinol oxidation-driven PCET to the FeS centre based on QM/MM free energy calculations.
Movie S3. Quinol oxidation-driven PCET to the heme b_L based on QM/MM free energy calculations.
Movie S4. Protonation dynamics of the fully reduced Q_i based on QM/MM MD simulations.
Movie S5. Protonation dynamics of the semiquinone Q_i based on QM/MM MD simulations.

SI References

Extended discussion: Functional relevance of mycobacterial adaptions at the Q_o site

To probe the functional relevance of the modular adaptations in the Q_{o1a} site, we studied redox tuning effects by *in silico* mutagenesis and electrostatic models (see Methods). In the D309N variant, the Rieske redox potential is downshifted by *ca*. 100 mV and heme b_L is upshifted by 50 mV relative to the WT model (Fig. 6f, Supplementary Table 5), whereas the menaquinone (MQ/SQ) has a predicted E_m of around -150 mV, *i.e.*, somewhat below its E_m value of -80 mV in membranes. These findings suggest that Asp309, which is replaced by a tyrosine in the canonical Complex III (Supplementary Fig. 20), could favour the electron transfer and increase the thermodynamic driving force for the menaquinone oxidation process. Protonation of His355 (by PCET from QH_2) increases the redox potential of the FeS Rieske centre by *ca.* 190 mV (+90 mV→+280 mV), leading to an effective proton-coupled redox potential of +120 mV (Fig. 6f). This protonation is favoured by the Asp309, which has an important redox-tuning effect on the Q_{o1a} site, and helps in establishing a downhill electron transfer between semiquinone and heme b_L, a process that is more favoured with the ubiquinol (+90 mV). The D309N substitution leads to a significant decrease in the hydration of the proton pathway next to the Rieske FeS centre, indicating that Asp309 could also support the proton release (Supplementary Fig. 5c).

The electron transfer between the quinone and heme b_L is linked to a proton transfer from the semiquinone ($QH^{\bullet}$) to Asp302 (Fig. 5b, d), which is adjacent to His110. Protonation of this site favours the closed conformation of the ion-pair between His110 and Asp302 (Supplementary Figs. 9e, 5d, e), whereas deprotonation of His110 leads to an ion-pair opening and increase of the hydration state of the proton pathway next to heme b_L (Supplementary Fig. 5d, e). The His110/Asp302 ion-pair is resolved in a closed conformation in our cryoEM structures (Fig. 3f), resembling the conformation observed prior to deprotonation of the quinol in our MD simulations (see Supplementary Fig. 5e)

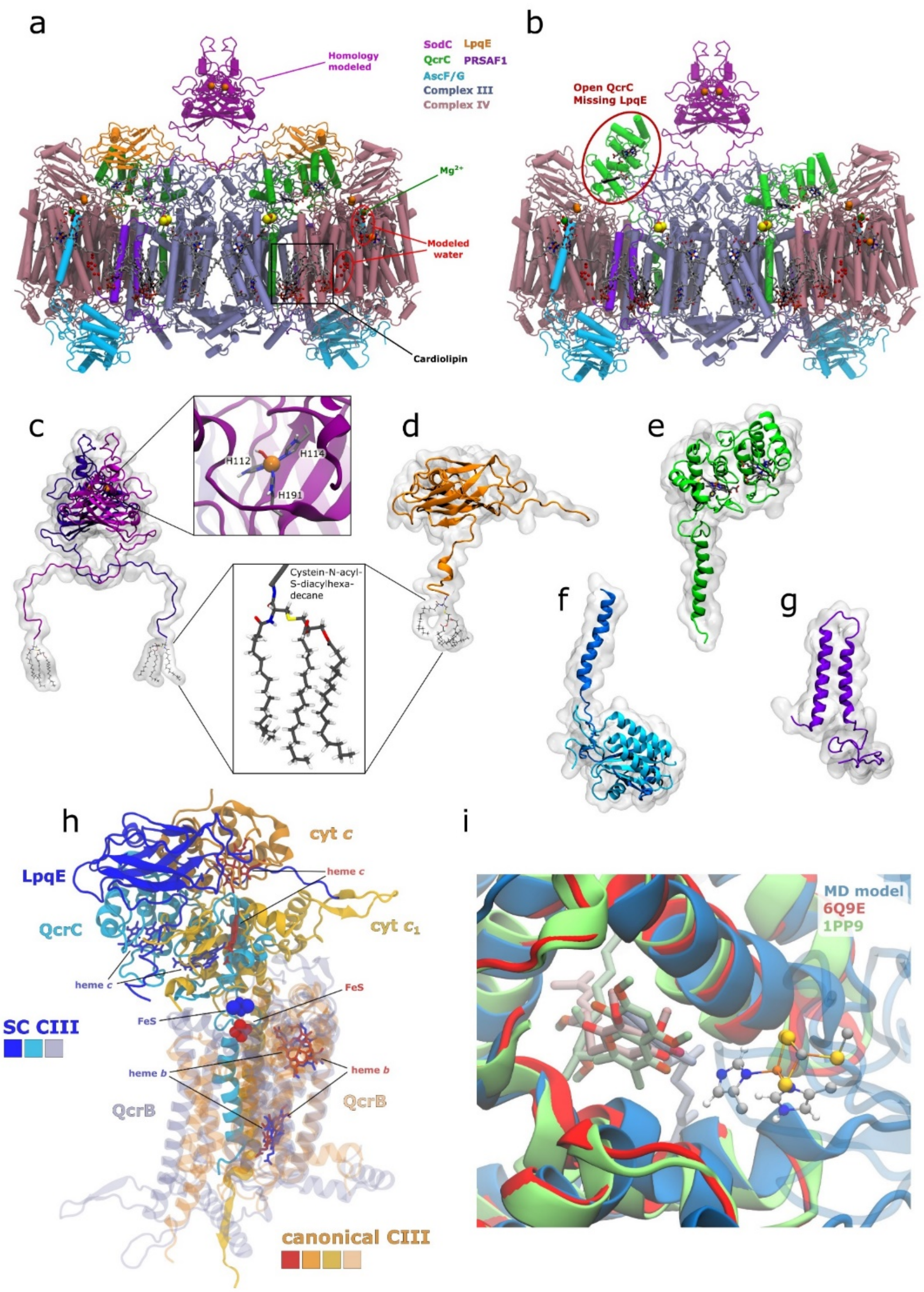

Figure S1. Details of the structural model of the SC (based on PDB IDs: 6HWH[1] and 6ADQ[2]). **a,** Symmetric model of the supercomplex showing modelled cardiolipins, as well as a SOD-C dimer (based on a homology model of PDB ID: 1PZS[3]). **b,** Asymmetric model of the supercomplex featuring one open QcrC and the absence of LpqE. **c,** The SOD-C dimer, containing a Cu ion in its active site, and featuring an N-terminal lipid modification anchoring it to the membrane. **d,** The LpqE subunit with the same lipid anchor as the SOD-C. **e,** The QcrC subunit, comprising two *c*-type hemes, which bridge the electron transfer chain between Complexes III and IV in the closed conformation. The open, disconnected conformation arises due to the absence of LpqE. **f,** The AscF/G subunits, located in the vicinity of the entrance of the D-channel in Complex IV, with a possible regulatory role. **g,** PRSAF1 (prokaryotic respiratory supercomplex association factor 1) subunit is located at the interface of Complexes III and IV. **h**, Overlay of the canonical *bc*₁ complex (in red colours; PDB ID: 1KYO[4]) with our current cryo-EM model (blue colours) aligned via the QcrB subunit (transparent). QcrC (cyan) occupies a similar position as cyt c_1 (yellow), but has heme c_1 in a different position, whereas LpqE (blue) partially overlaps with the bound cyt *c* (orange). The FeS cluster is shifted closer to the Q_o site in the SC as compared to the canonical complex, where the Rieske protein is mobile. **i,** The quinol was modelled in the Q_{o1a} site based on structural data of quinol and stigmatellin binding in canonical Complex III (PDB IDs: 6Q9E[5], 1PP9[6], *c.f.* Methods). The predicted binding site Q_{o1a} in the supercomplex (in blue) in comparison to ubiquinone (in red) and stigmatellin (in green) from the canonical Complex III.

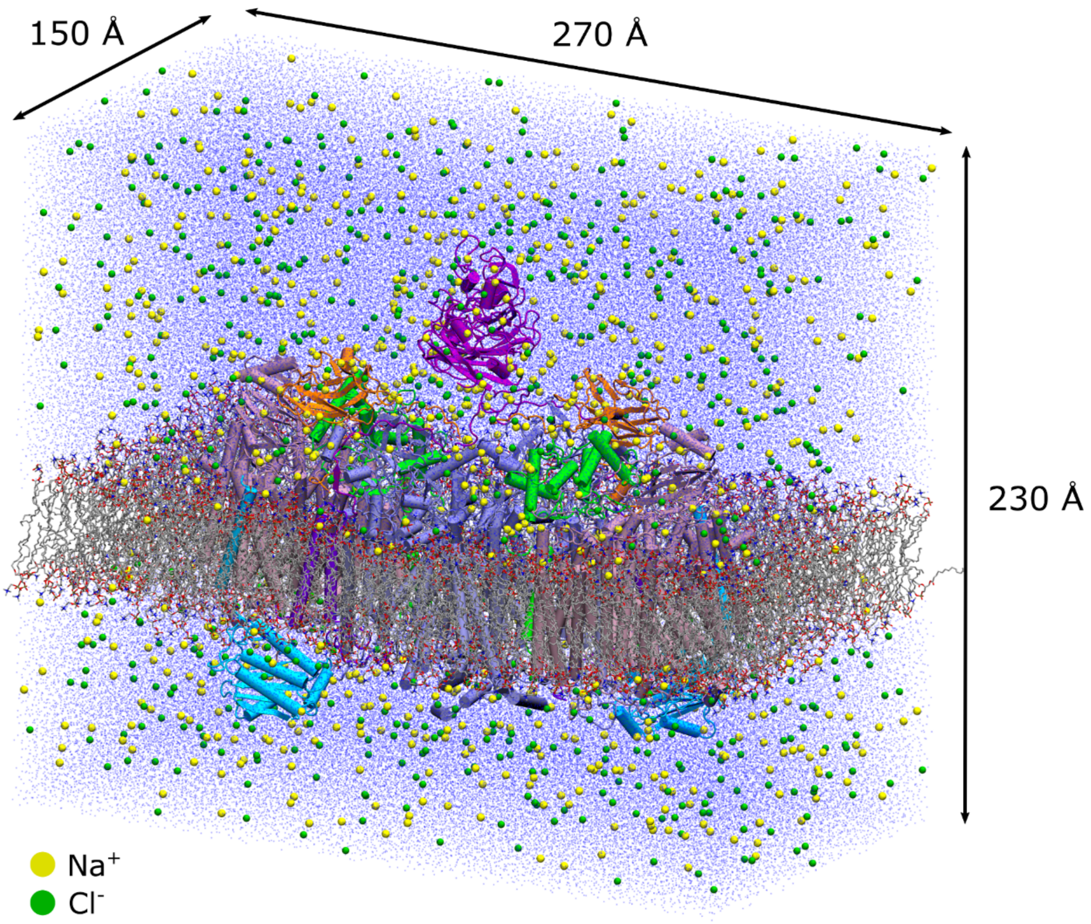

Figure S2. Molecular dynamics setup of the *Mycobacterium smegmatis* III_2IV_2 supercomplex. The protein is embedded in POPC lipid membrane and solvated in a waterbox and neutralised with 150 mM NaCl. The protein is shown in cartoon representation, the lipids as sticks, the ions as spheres, and water molecules as blue dots. The MD system comprises *ca*. 930,000 atoms. See Tables S1-S3 for simulation setup details.

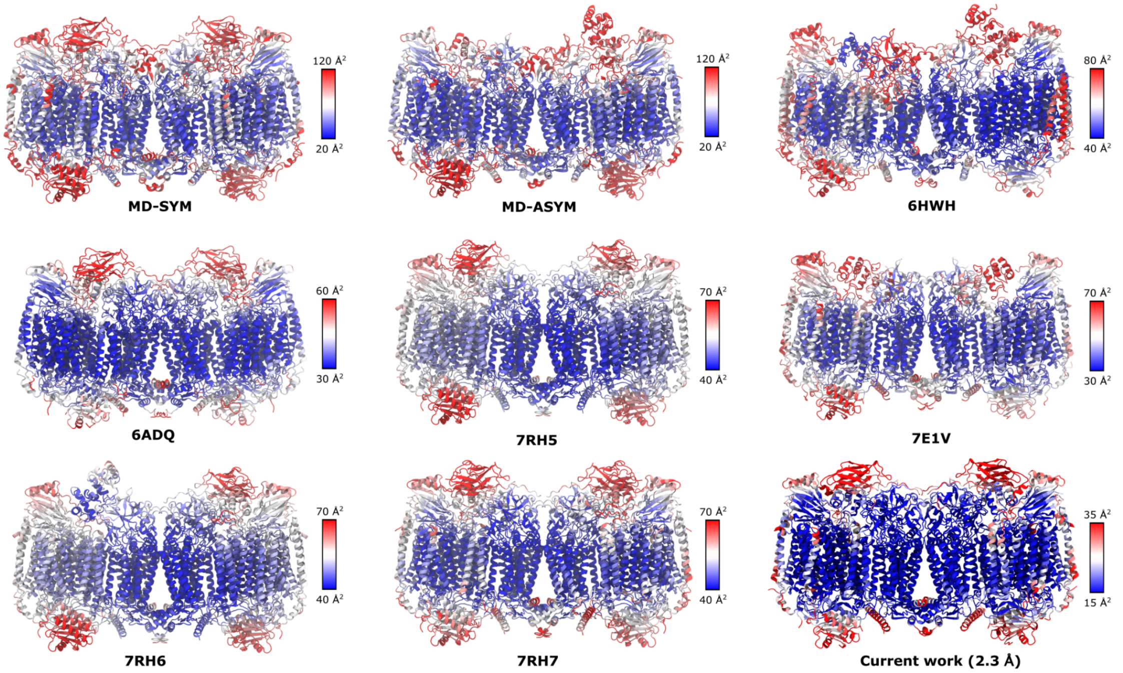

Figure S3. **B-factors calculated from MD simulations and from cryoEM experiments.** The simulations show an overall similar B-factor distribution in the different models. The dynamics of SOD-C was not included in this analysis to its high flexibility. LpqE and AscF/G show a high flexibility in the symmetric structure, while the asymmetric structure has an open QcrC conformation and is dynamically quite mobile. The membrane subunits show a high rigidity. See Ref.[1,7-9] for other cryoEM models.

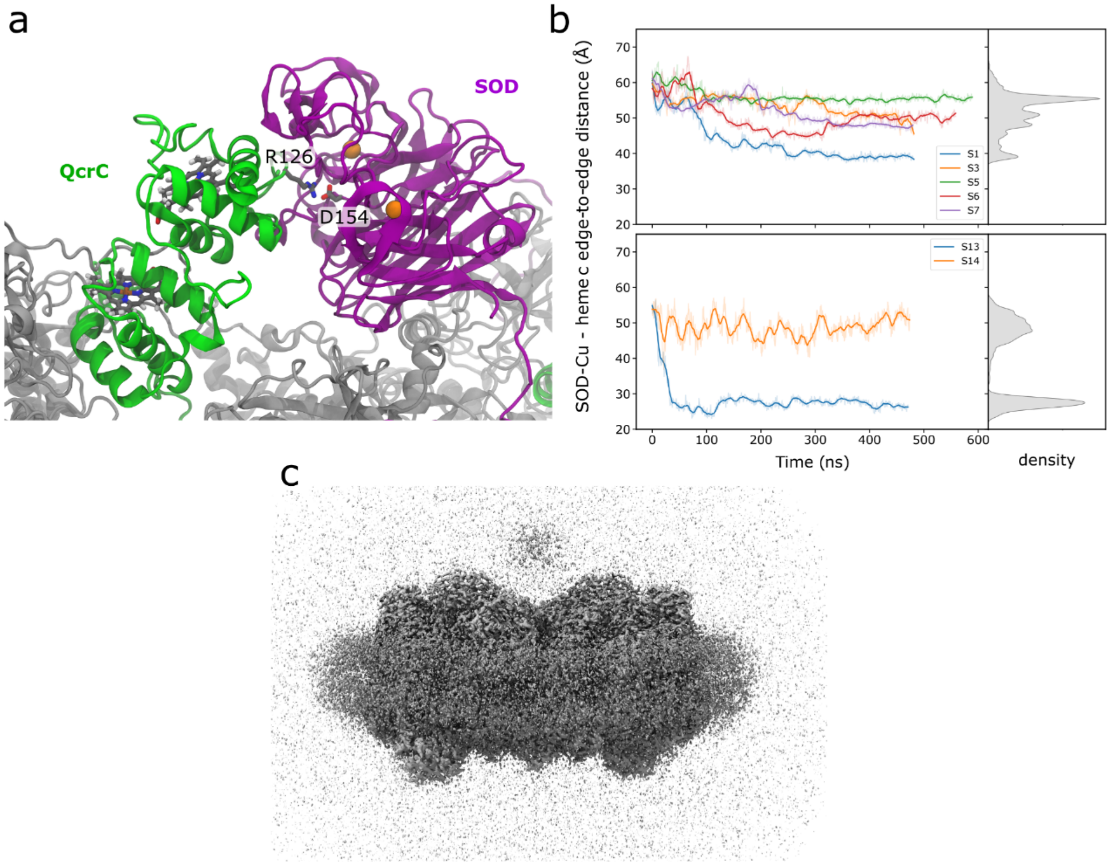

Figure S4. SOD-C interaction and dynamics. a, SOD-C forms contacts with the QcrC subunit in simulation S13, by establishing ion-paired contacts between the subunits, in addition to unspecific non-polar contacts. **b,** *Edge-to-edge* distances between the SOD-C copper and heme *c* of QcrC (*top*: symmetric model, *bottom*: asymmetric model). Only the shortest distance is plotted. The distribution suggests that SOD-C does not form specific contacts with the supercomplex but sample many non-specific conformations, consistent with the blurred cryoEM densities. Shaded lines represent raw data while the solid lines show the smoothed data. **c,** SOD-C shows a blurred density map, consistent with the high mobility of the subunit in MD simulations. Density shown at a threshold of 0.22.

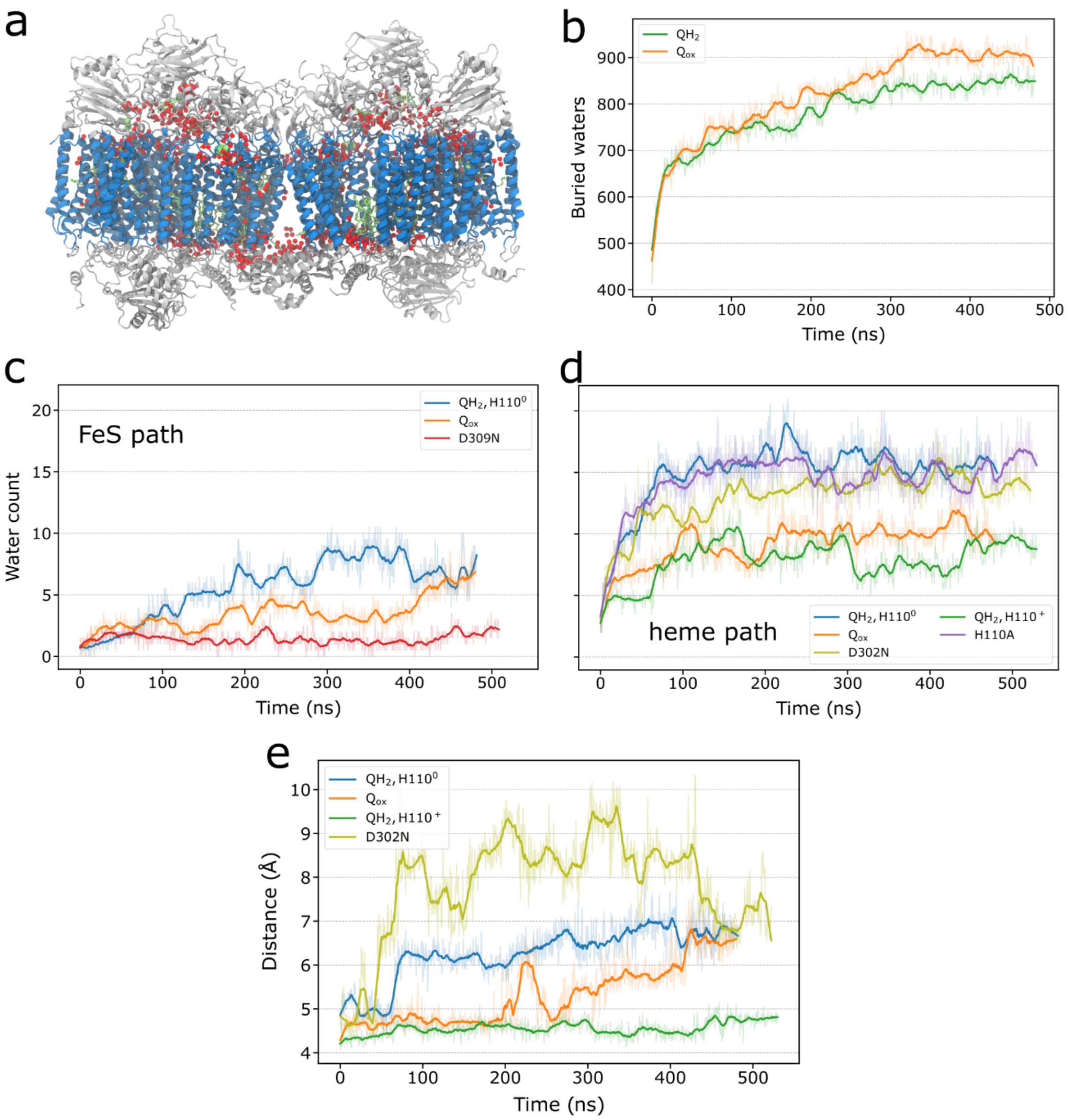

Figure S5. Dynamics of buried water molecules in the SC. a, Snapshot of buried water molecules in the membrane plane and in the vicinity of the cofactors (simulation S1, Supplementary Table 1). Parts of the protein that are located in the membrane plane are coloured in blue. Waters are shown as red spheres and cofactors or cardiolipin as light green sticks.. **b,** Simulations (S1/S7, Supplementary Table 1) show rapid increase in buried water molecules during the first 50 ns and reach a plateau after *ca*. 300 ns. **c, d,** Hydration levels in the putative proton exit pathways, leading from the Q_{o1a} site to the P-side bulk. The D302N mutation results in a significant decrease in the hydration level at the Q_{o1a} site. The hydration along the heme pathway depends on the interaction between Asp302 and His110 in the MD simulations, resulting in lower hydration levels in the closed ion-pair conformation (see panel e). **e,** The His110-Asp302 distance during MD simulations, show a stable salt-bridge when His110 is modelled in a protonated state, and a more dynamic hydrogen-bond when Asp302 or His110 are neutral. The D302N mutation and the neutral His110 state result in dissociation of the residues during the MD simulations. Raw hydration data is shown is shaded lines, while the smoothed data is shown in solid lines.

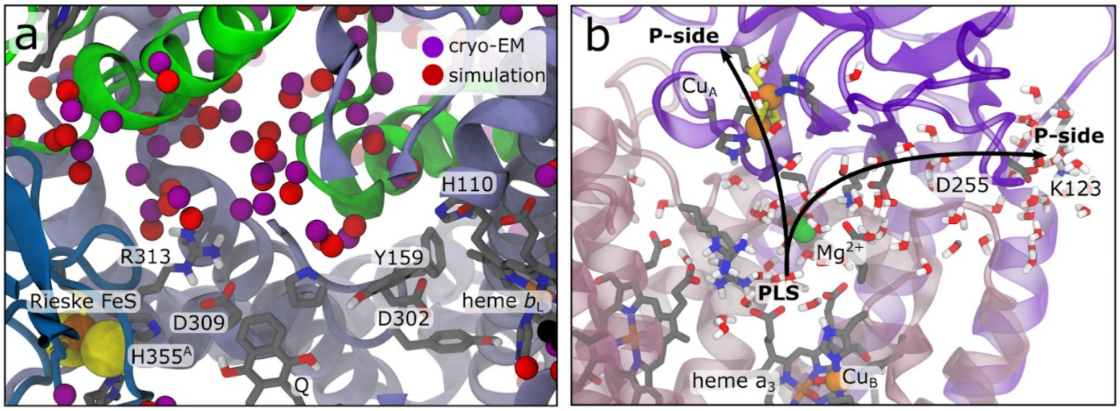

Figure S6. Comparison of MD and cryoEM water molecules. a, Comparison of experimentally resolved water molecules from cryoEM (in purple) with water molecules predicted by MD simulations (in red). The figure shows water molecules from the last snapshot of simulation S1 that are within 2 Å of an the experimentally identified water molecules. The MD simulations and cryoEM predict overall highly similar water positions. **b,** Proton pathways in Complex IV, leading from the proton loading site above heme a_3 to the P-side of the membrane via the Mg^{2+} ion. One output pathway leads to the P-side close to the Cu_A site, while the other pathway bridges D255 and K123 of subunit II to the P-side.

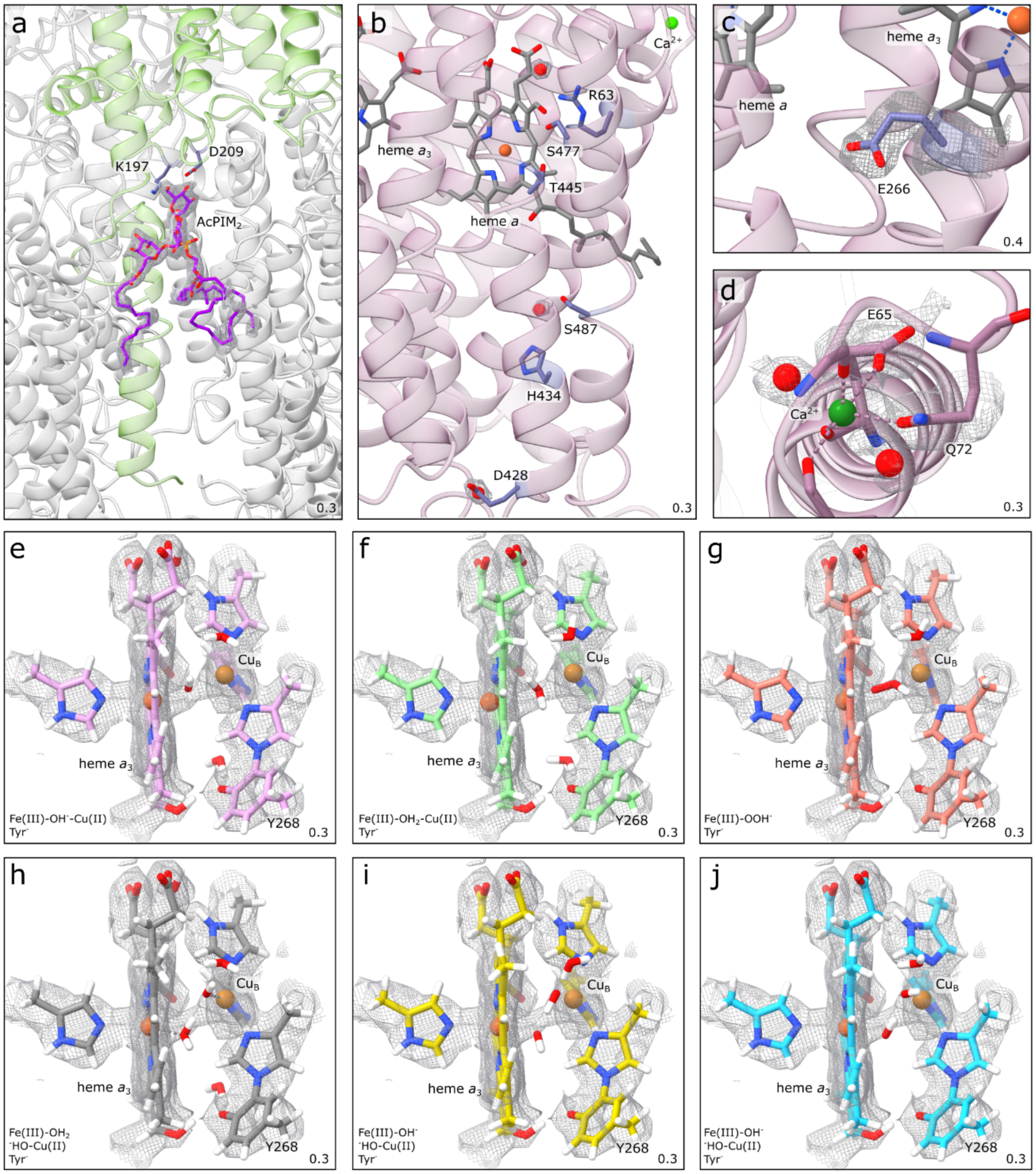

Figure S7. Example cryoEM densities and BNC models based on the 2.3 Å map. a, An $AcPIM_2$ lipid is found next to the top of the QcrC helix, in close proximity of Lys197 and Asp209, suggesting its structural importance. **b,** The H-channel region of the supercomplex is conserved, but barely hydrated compared, as further corroborated by MD simulations, implying that the region does not conduct protons. **c,** Glu266 at the end of the D-channel, resolved in a downwards conformation. **d,** A Ca^{2+} ion in Complex IV coordinated by Glu65 and Gln72, three backbone oxygens and two water molecules, modelled with 0.5 occupancy, is located 12 Å heme *a*. **e-j,** Geometry optimised QM models based on the cryoEM map, with different ligands modelled in the density between the Fe^{3+} and Cu^{2+}.

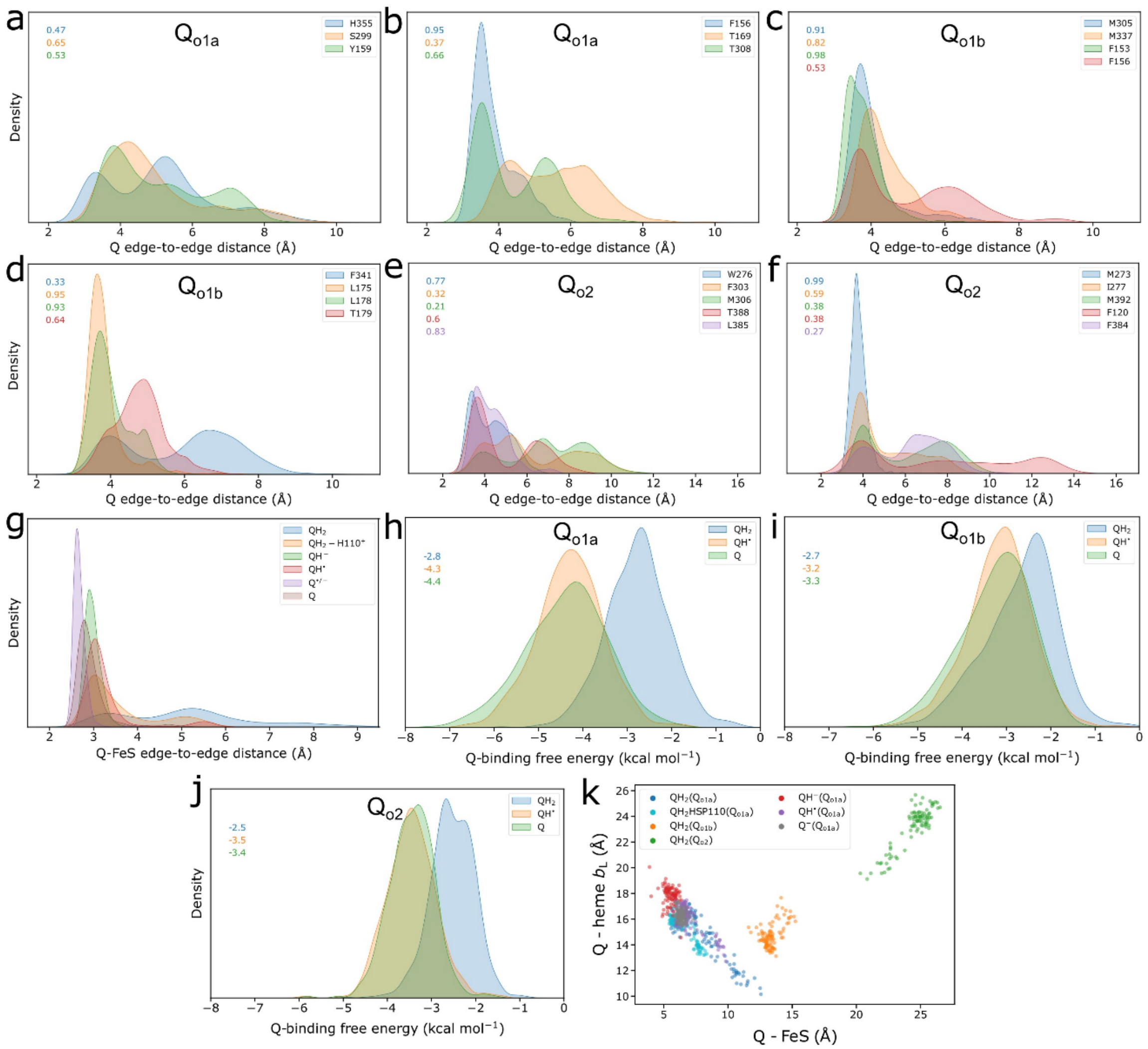

Figure S8. Quinone dynamics in the Q_o sites. a-f, *Edge-to-edge* distance distributions between Q in the different Q sites and nearby residues. Only residues within 4 Å of Q in at least 20% of the MD trajectory are shown. The fraction of contacts is reported on the top left of the figure. **a, b,** The Q in Q_{o1a} forms hydrogen-bonded contacts with His355 and Tyr159, as well as other non-specific polar and aromatic residues. **c, d** The Q in Q_{o1b} forms π-stacking interactions with Phe153, as well as non-polar interactions with several other residues. **e, f** The Q in Q_2 forms π-stacking interactions with Trp276, as well as with other non-polar residues. **g,** The *edge-to-edge* distance between Q and the FeS Rieske centre depend on the redox state of the quinone. The menaquinone binds in a stable binding pose in Q_{o1a}, especially in all anionic charged states, although transient unbinding events (distance > 3.5 Å) can be observed. Protonation of His110 stabilises the quinone binding. **h-j,** Estimates of binding free energies (from PBSA-MM calculations) of QH_2, $QH^•$, and Q in the different sites calculated at ε=10. Similar affinities are observed for all three sites, but with a somewhat overall stronger binding in the Q_{o1a} site relative to Q_{o1b} / Q_{o2}. **k,** Distance map showing the Q position relative to the FeS and heme b_L cofactors for different trajectories. In one trajectory (dark blue), quinol is dynamically more flexible, and dissociates from Q_{o1a} close to Q_{o1b}.

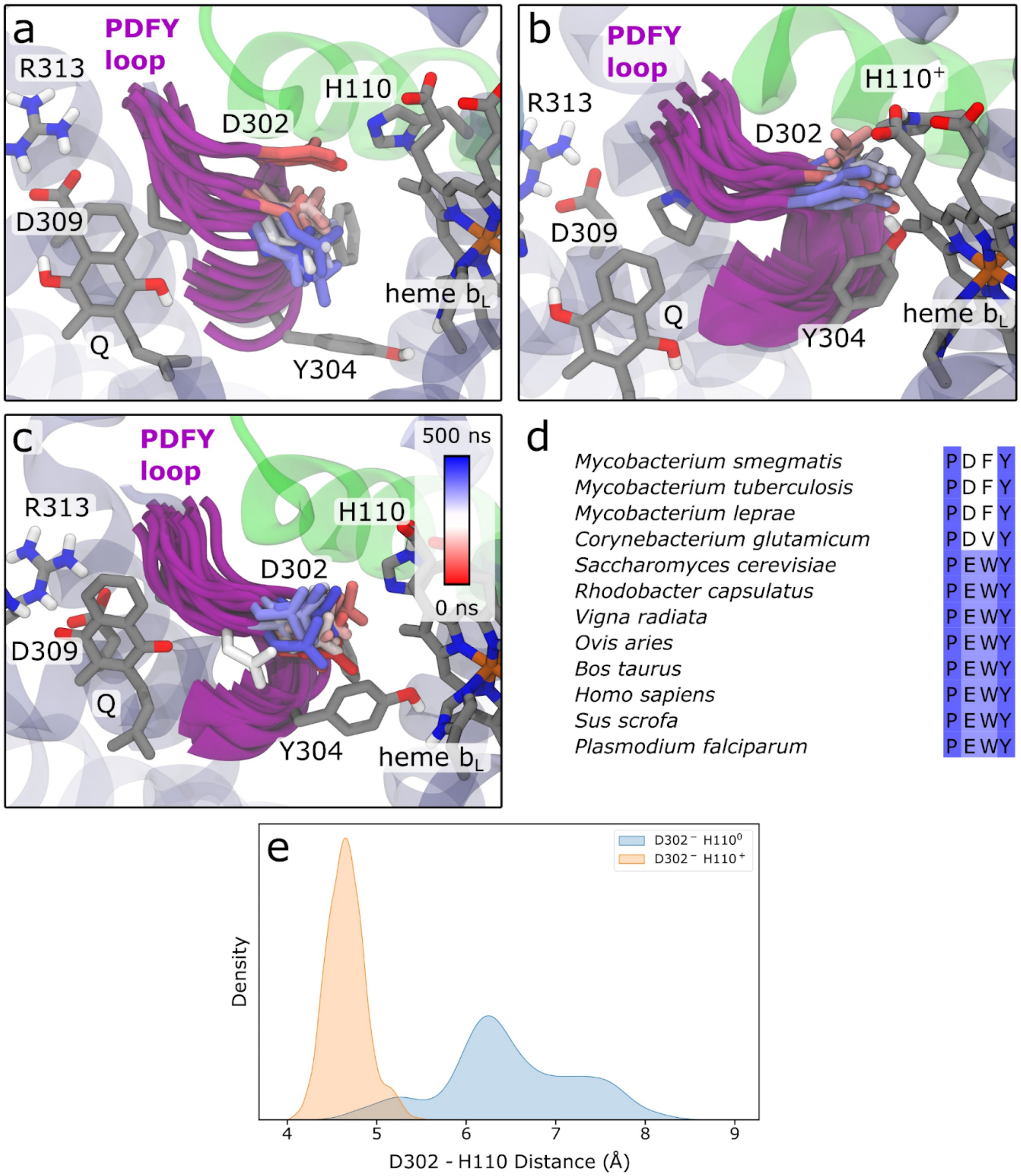

Figure S9. Dynamics of the PDFY loop in the Q_{o1a} site. Data from MD simulations S1 (**a**, QH_2), S2 (**b**, QH_2, protonated H110), and simulation S7 (**c**, oxidised Q). The PDFY loop is shown in purple. The Asp302 conformations are coloured by the simulation time (red to blue). **d,** Sequence conservation of the PDFY loop across different species. **e,** In simulations with a protonated His110 (simulations S2, S9, S12), the residue forms a stable ion-pair with Asp302, whereas Asp302 is dynamically highly flexible in the absence of the ion-pair (neutral His110, S1, S3, S5).

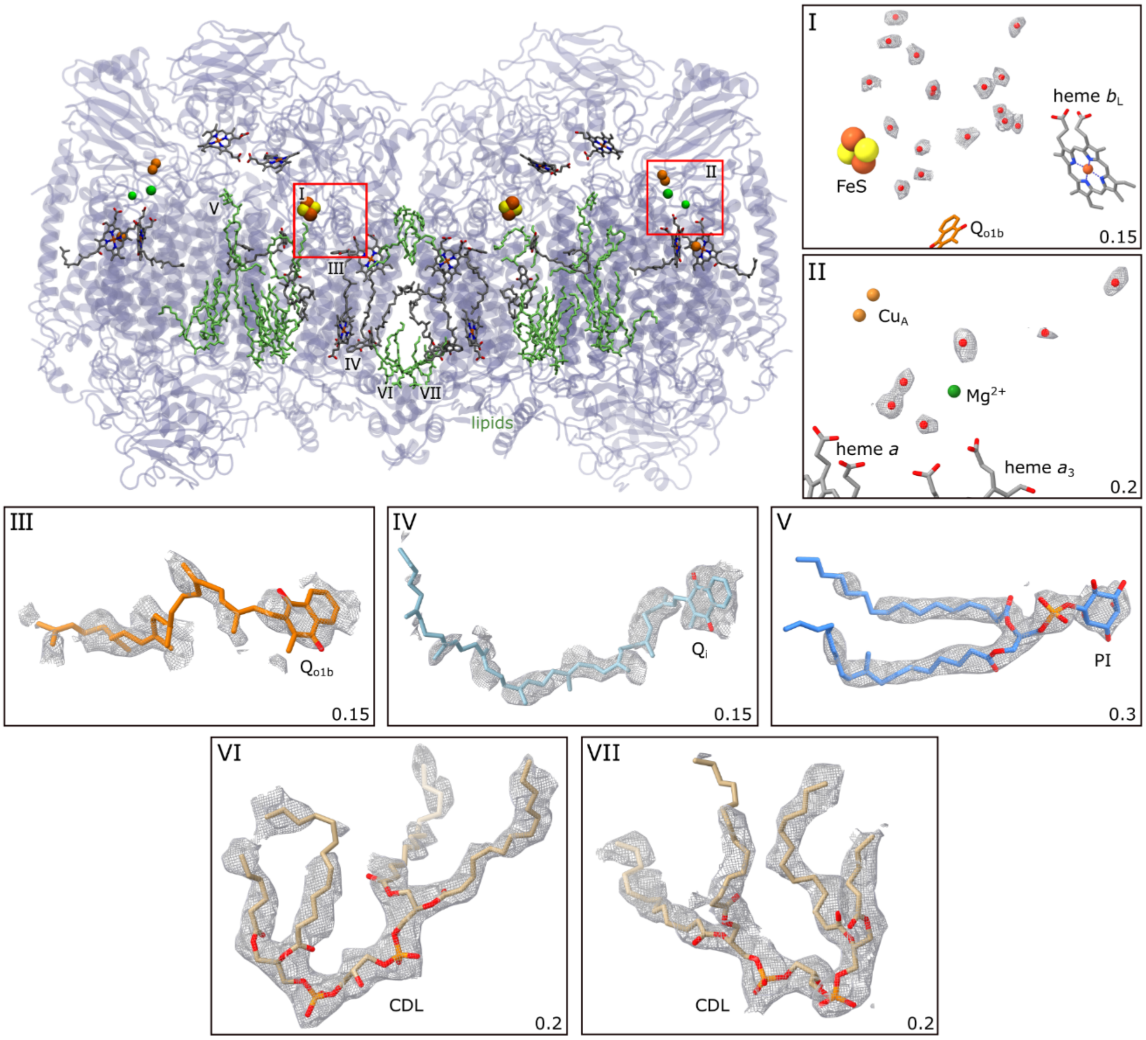

Figure S10. Example cryoEM densities of key regions based on the of the 2.8 Å map model. The figure shows water molecules around the Q_{o1a} site; the menaquinone and cardiolipin molecules at the Q_i site; and resolved water molecules around propionate region of heme a_3. Waters are show as red spheres.

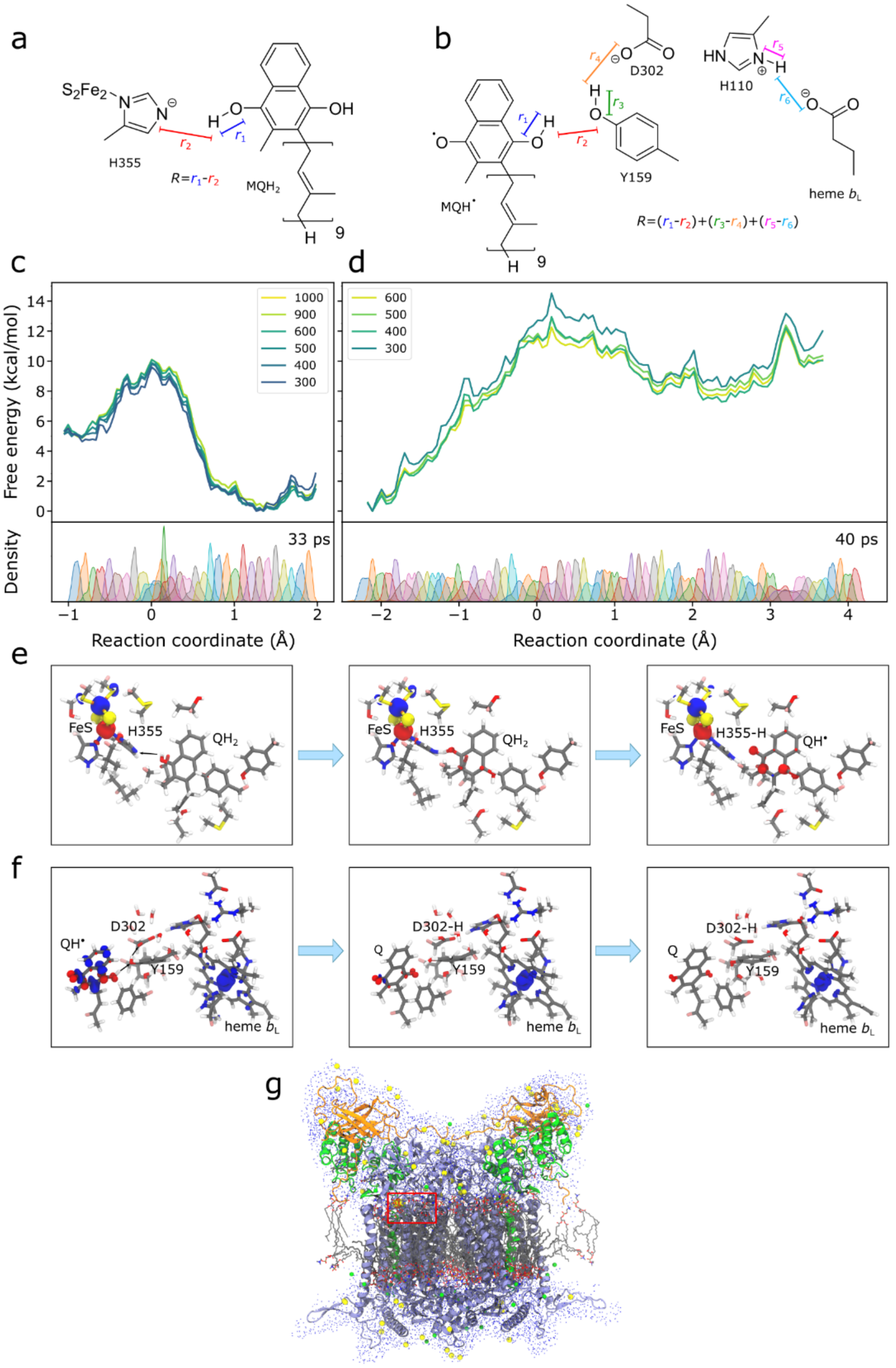

Figure S11. QM/MM free energy simulations of the PCET reactions in the Q_{o1a} site. The QM/MM reaction coordinates were defined as a linear combination of bond-breaking and bond-forming distances for **a,** the first PCET from menaquinol to the Rieske FeS-centre and **b,** the second proton transfer from menaquinol to Asp302 coupled to electron transfer to heme b_L. **c, d,** Convergence (top) and overlap (bottom) of the free energies for **c,** the first and **d,** the second PCET step. **e, f,** Spin densities for the reactant, transition, and product state of **e,** the first and **f,** the second PCET step. The densities are shown as red and blue volumes. **g,** Overview of the QM/MM simulation system. The location of the QM region is indicated by the red rectangle.

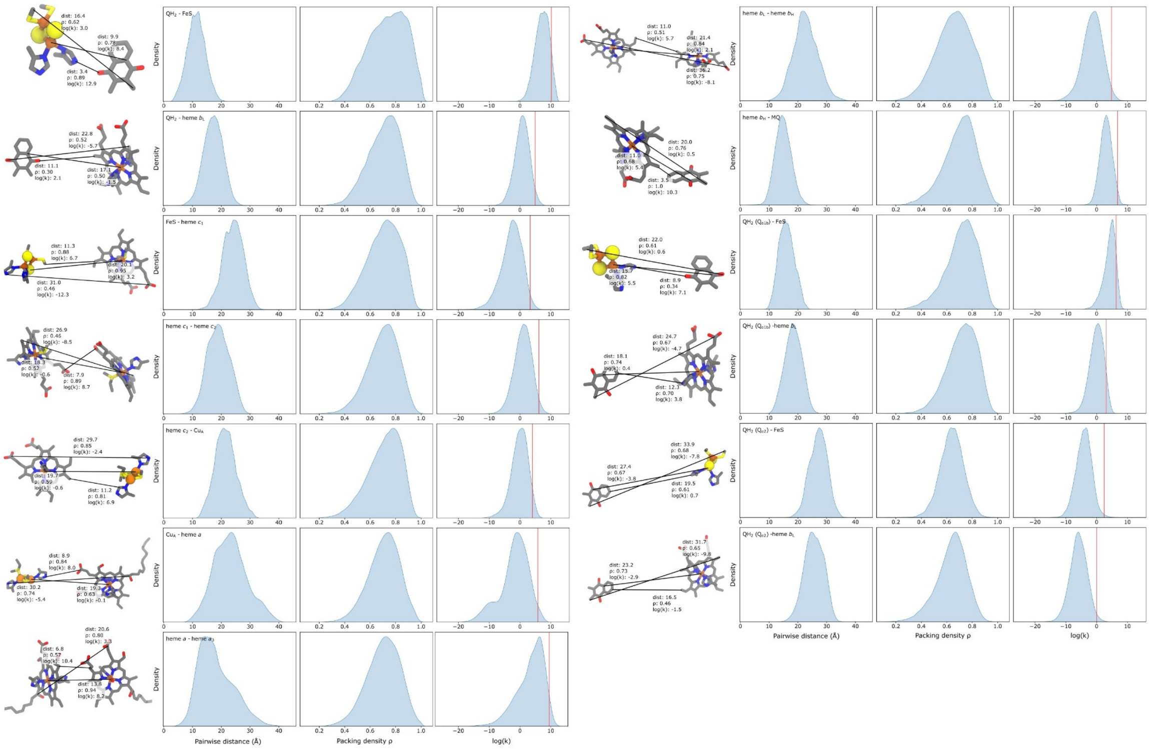

Figure S12. Details of the electron transfer rate calculations. For each donor-acceptor pair, the packing densities and electron rates were calculated along all possible donor-acceptor pair, by considering the explicit intervening protein surrounding between the groups (see Methods). *Left*: Snapshot of the donor-acceptor groups with example distances (*edge-to-edge*, *mean*, and *max* distance). *Right:* Distribution of pairwise distances, packing densities (ρ) and calculated electron transfer rates (*k*). The average electron transfer rate, calculated as sum of the coupling-weighted rates, is marked with a red line.

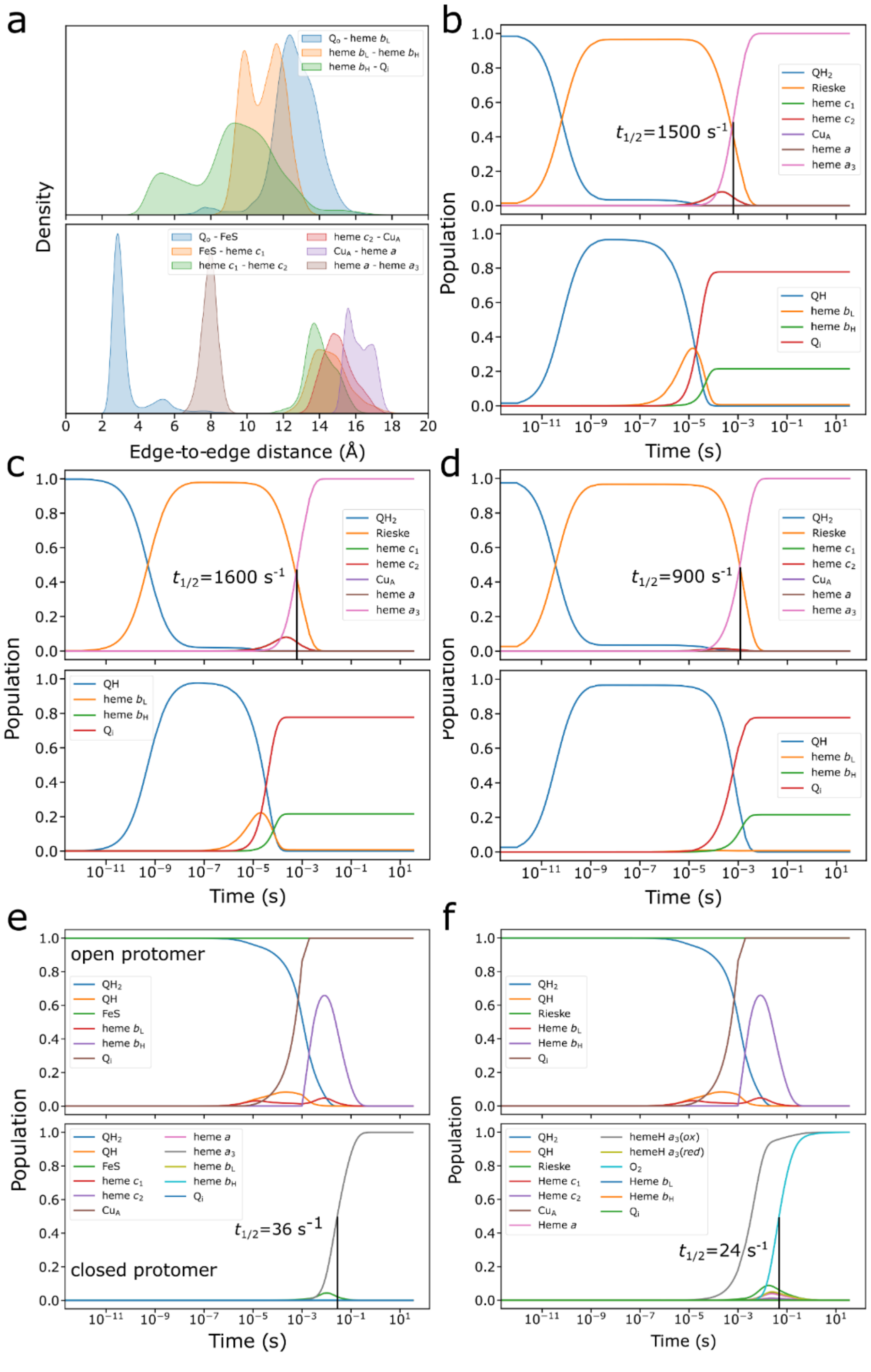

Figure S13. Kinetic models of electron transfer in the supercomplex. a, *Edge-to-edge* distance distributions from MD simulations S1, S3, S5, S6, and S7 (symmetric states). All predicted rates are within physiologically relevant timescales for the electron transfer. **b,** Kinetic model of the symmetric complex with the terminal electron transfer from Q_{o1a} to heme b_L, modelled as an irreversible step. **c,** Model of the electron transfer using Q_{o1a}→Rieske and Q_{o1a}→heme b_L rates calculated from the QM/MM barriers based on transition state theory. **d,** Electron transfer in the closed QcrC protomer of the asymmetric supercomplex. **e,** Electron transfer kinetics for electron “leakage” from the open to the closed protomer, shows a much slower electron transfer rates as compared to the closed protomer. **f,** Electron “leakage” model accounting for the 4 ms proton uptake via the D-channel

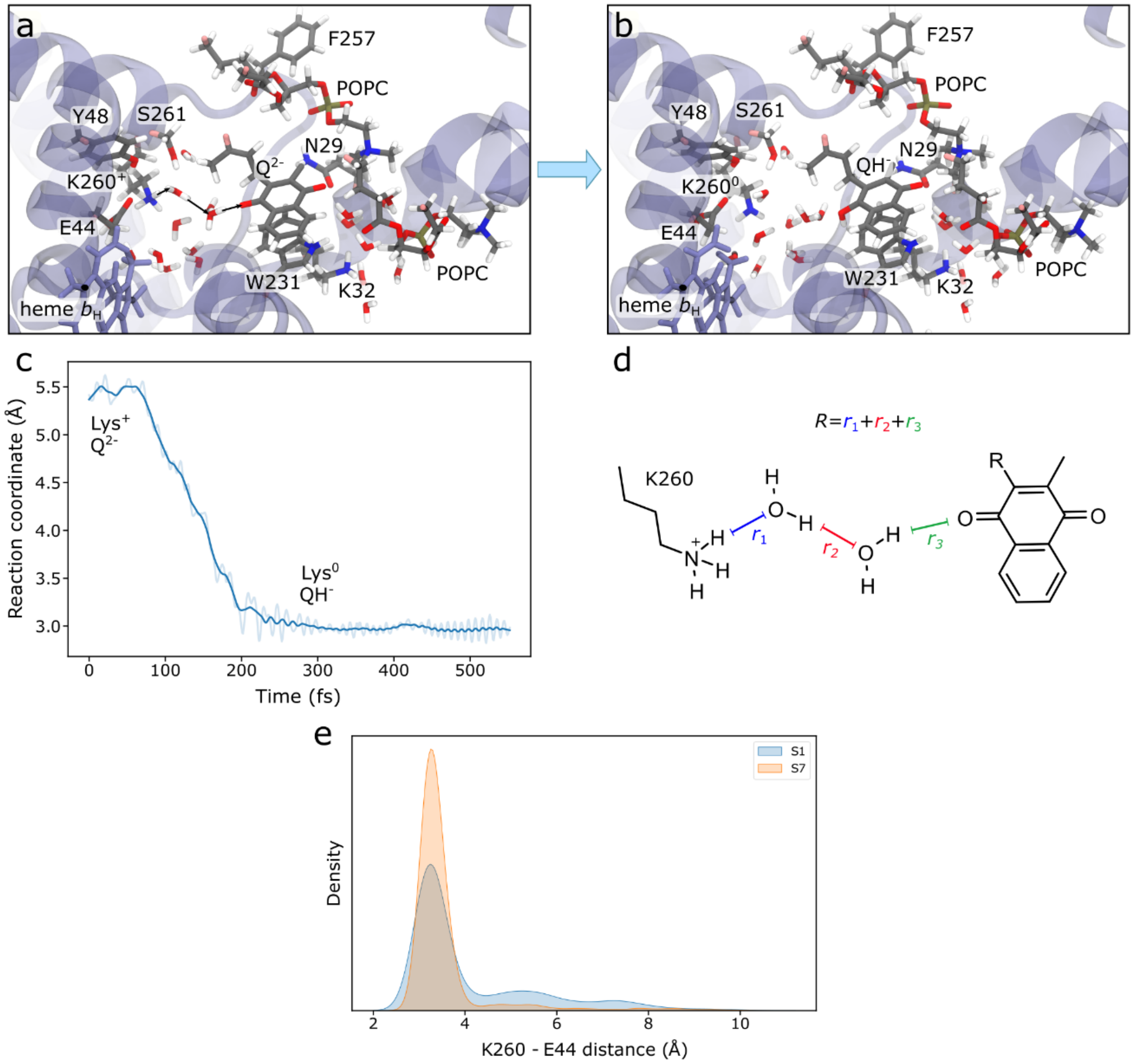

Figure S14. QM/MM dynamics at the Q_i site. a, b, QM/MM molecular dynamics simulations, show proton transfer from nearby Lys260 to the quinone in Q_i upon reduction to Q^{2-}. **c,** The proton is transferred from Lys260 to Q^{2-} on picosecond timescales in unbiased QM/MM MD simulations. The simulation was started from a classically relaxed MD snapshot with the hydrogen-bonded network well-aligned towards the quinone. The shaded line shows the raw data, while the solid line is the smoothed data. **d,** Schematic representation of the reaction coordinate shown in panel **c**. **e,** The Lys260 - Glu44 ion-pair samples transient open conformation that could allow for proton transfer from the Lys260 to the quinone.

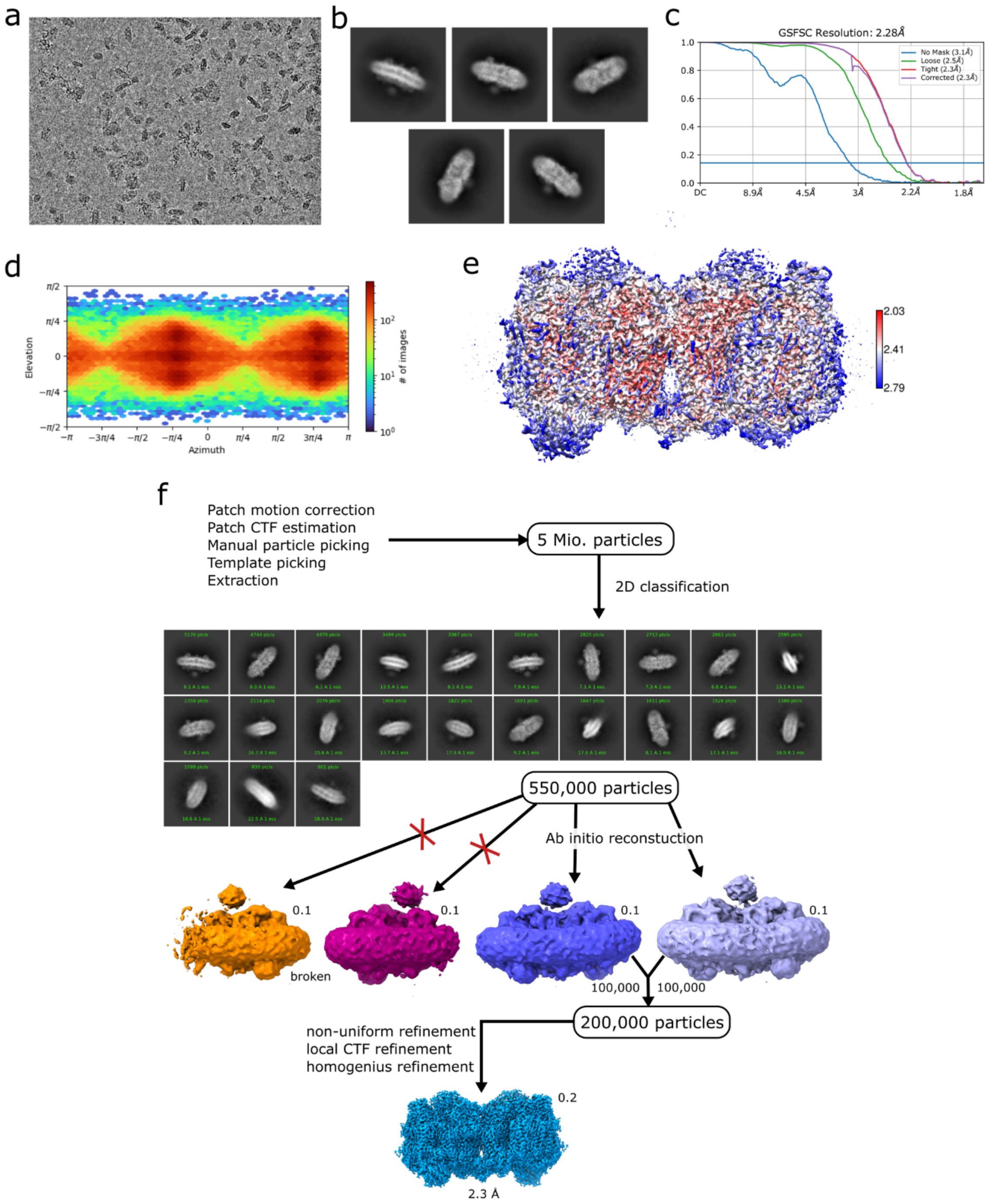

Figure S15. CryoEM data analysis and validation of the 2.3 Å map. a, Example of micrograph showing particle orientation and sample homogeneity. **b,** Example of the 2D-class averages with views of different particle orientation. **c,** Fourier shell correlation (FSC) curve of the final 3D-reconstruction. **d)** Viewing direction distribution of particles. **e,** Estimation of the local resolution. **f,** Classification tree and particle sorting approach.

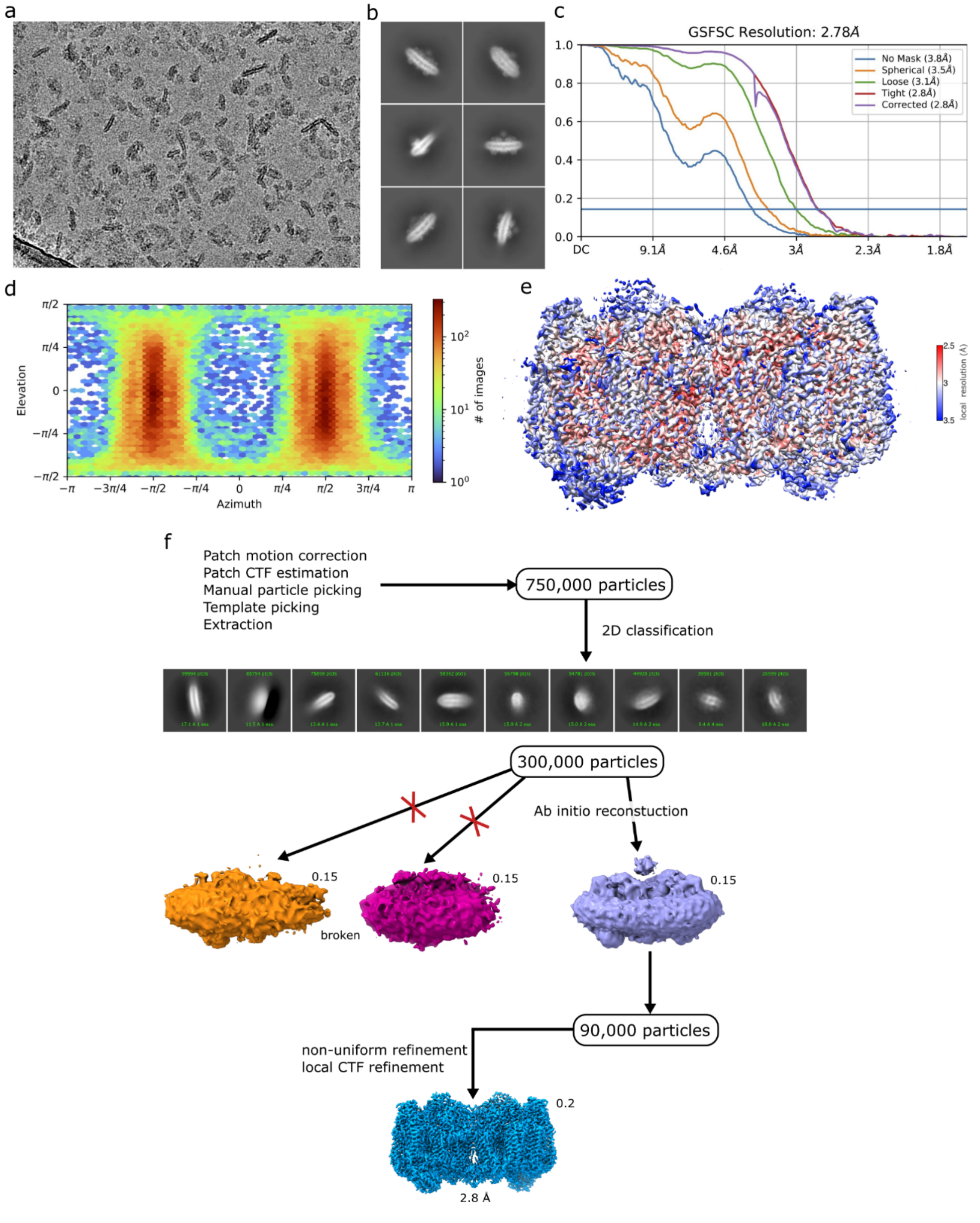

Figure S16. CryoEM data analysis and validation of the 2.8 Å map. a, Example of micrograph showing particle orientation and sample homogeneity. **b,** Example of the 2D-class averages with views of different particle orientation. **c,** Fourier shell correlation (FSC) curve of the final 3D-reconstruction. **d)** Viewing direction distribution of particles. **e,** Estimation of the local resolution. **f,** Classification tree and particle sorting approach.

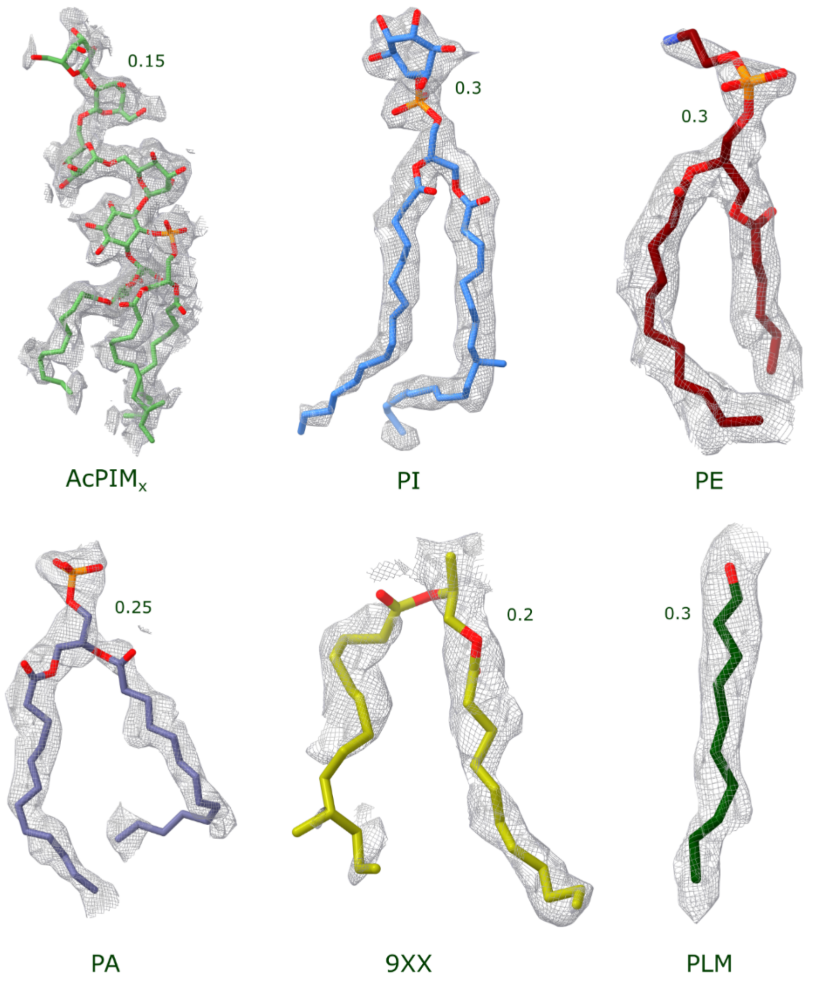

Figure S17. Example cryoEM densities of lipid modelled based on the of the 2.3 Å map model. **AcPIM**: acylated phosphatidylinositol mannoside, with the density supporting modelling of at least five mannoside sugars. **PI**: Phsopatidylinositol. **PE**: phosphatidylethanolamine; **PA**: phosphatidic acid; **9XX**: (2S)-1-(hexadecanoyloxy)propan-2-yl(10S)-10-methylocta-decanoate; **PLM**: palmitic acid.

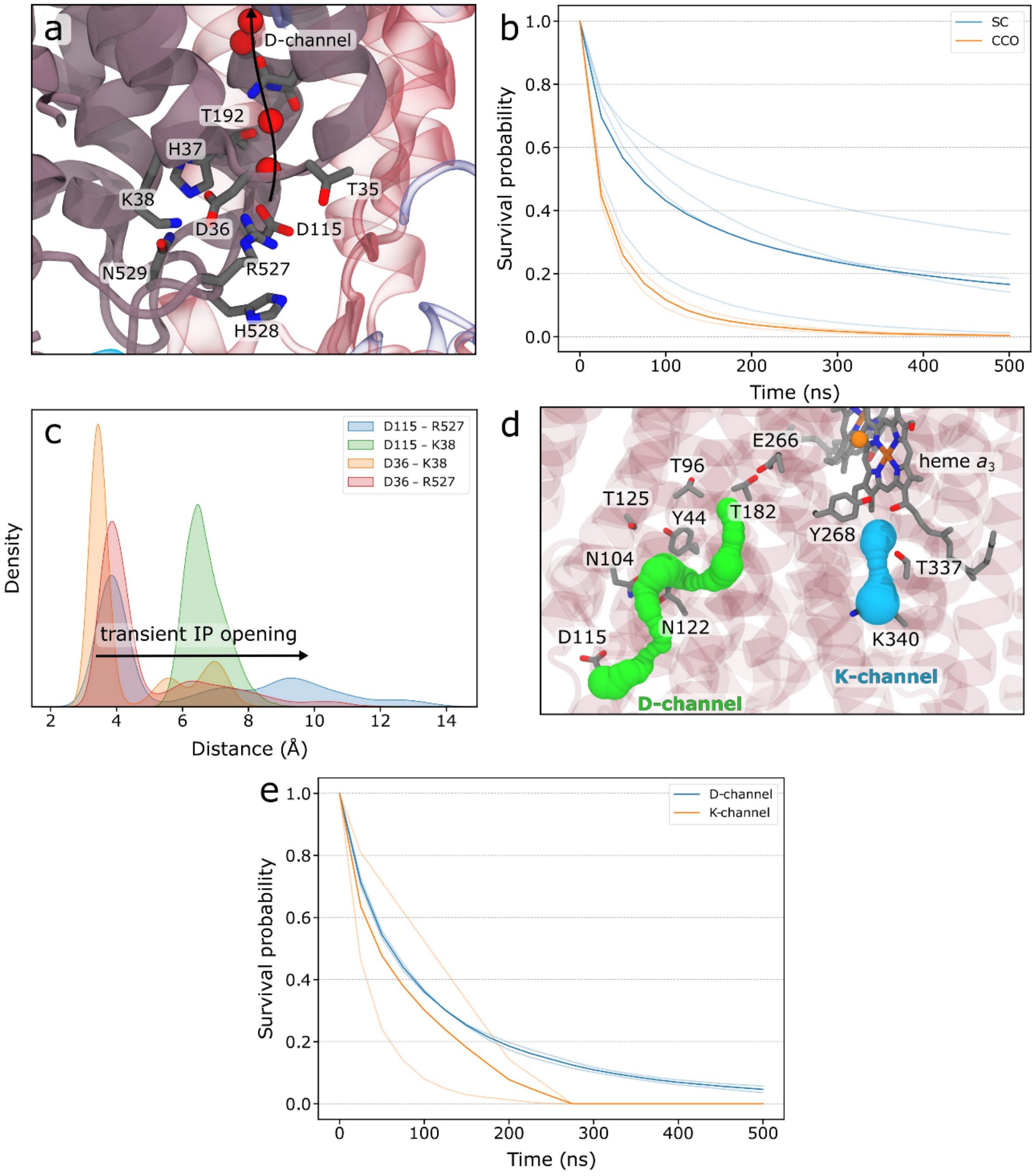

Figure S18. Hydration dynamics in the D- and K-channel. a, Charged and polar residues at the entrance of the D-channel. **b,** Survival probability of water molecules at the entrance of the D-channels (simulations S1, S7, S17+18, Table S1). The canonical Complex IV (CCO, bovine) exhibits fast water exchange with the bulk ($t_{1/2}$=23 ns), while the supercomplex (SC) shows a significantly longer water exchange rate ($t_{1/2}$=70 ns). **c,** Ion-pair distances between Asp115-Arg527, Asp115-Lys38, Asp36-Arg527 and Asp36-Lys38 at the D-channel entrance. The ion-pairs remain mostly closed, but transient opening conformations are observed. **d,** Comparison of the volumes of the D- and K-channels, estimated using CAVER3[10]. The D-channel has nearly three times the volume of the K-channel (134 Å^3 vs 47 Å^3, respectively). **e,** Water survival probabilities in the D- and K-channel (see Methods) suggest that water molecules have a higher mobility in K-channel, and could rationalise why less water is resolved in the K-channel in the experimental structures of Complex IV. Shaded lines show the individual channels, while the solid line represent the average between them.

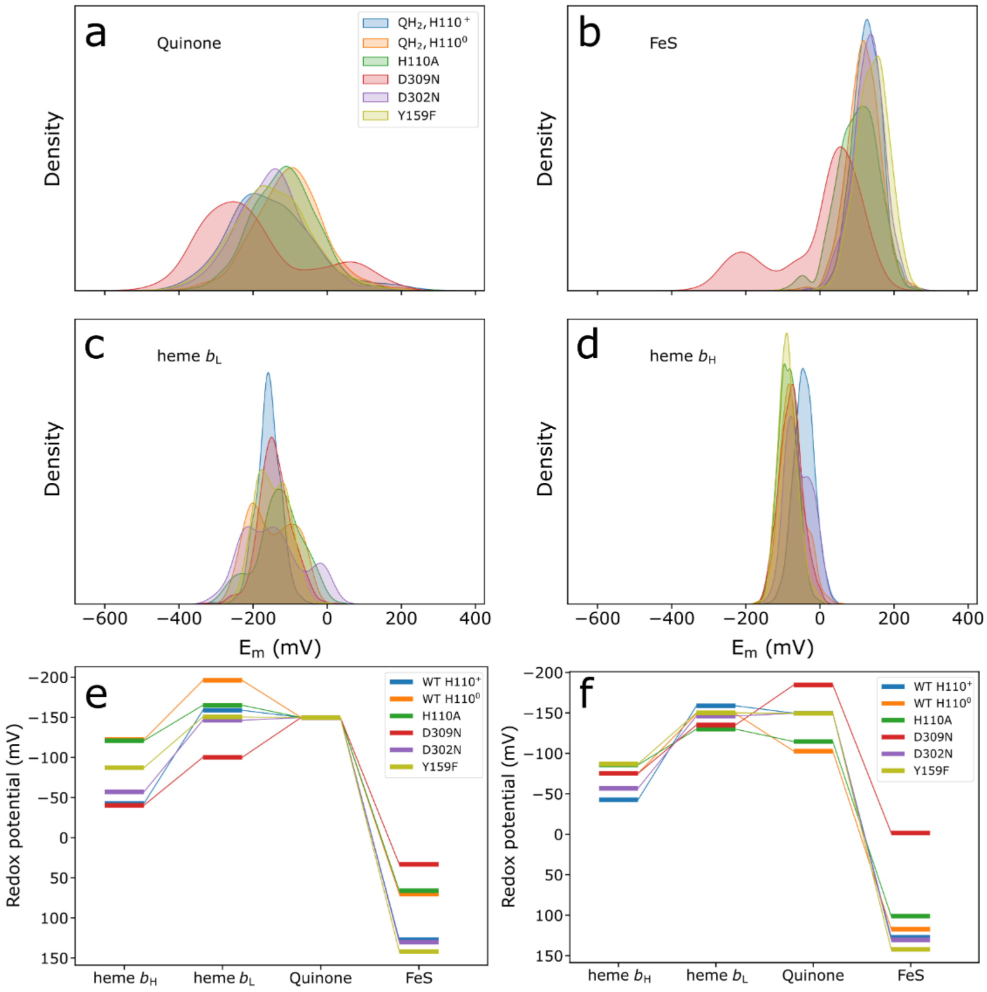

Figure S19. Computed redox potentials around the Q_{o1a} site. a-d, Calculated redox midpoint potentials from simulations of wild type SC (with and without protonated His110), as well as for respective *in silico* mutants (with His110 protonated). **e,** MD-averaged redox potentials relative to the menaquinone potentials and **f,** MD-averaged absolute redox potentials.

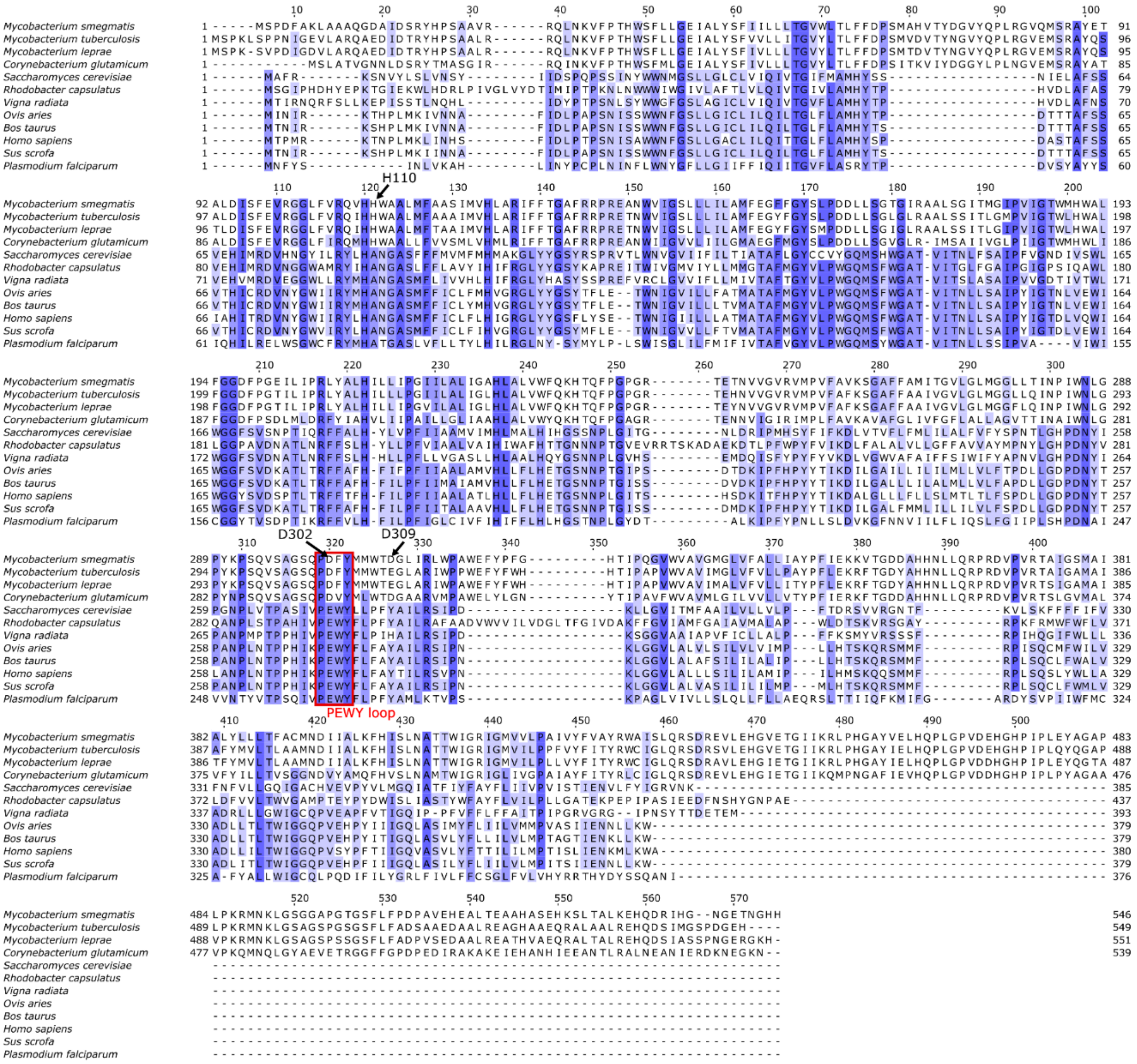

Figure S20. Multiple sequence alignment of QcrB. The analysis was performed using Clustal Omega[11].

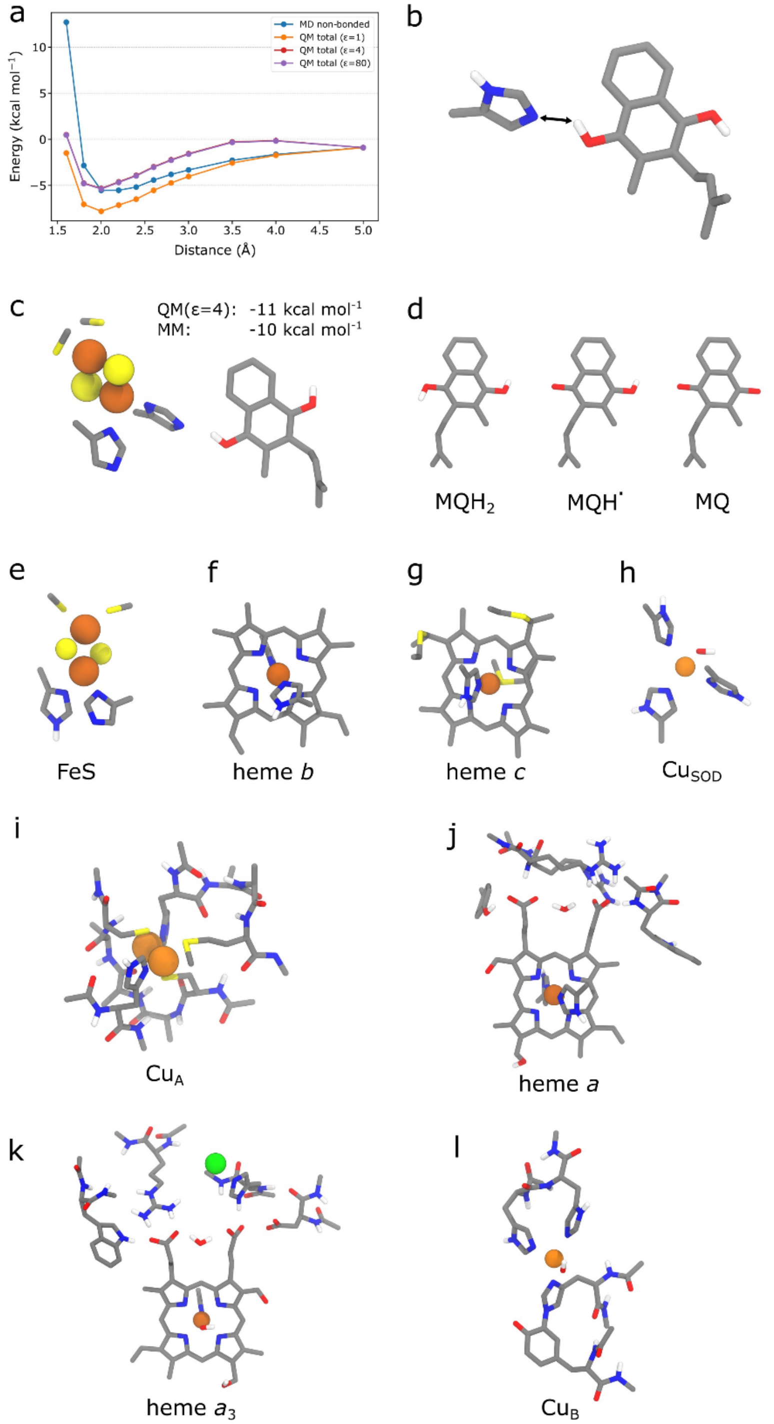

Figure S21. Forcefield benchmarking and parameterisation. a, b, The validity of the quinone parameters was benchmarked against DFT calculations (QM level: B3LYP-D3/def2-TZVP). MQ and His coordinates were independently optimised in vacuum and interaction energies were calculated at both QM and MM level at specific distances. QM interaction energies were also calculated with dielectric constants of 1, 4, and 80. **c,** Binding energies between MQ and the FeS centre resulted in similar interaction energies: (-11 kcal mol^{-1} QM model; -10 kcal mol^{-1} MM model). **d-l,** Cofactor models used for the force field parameterisations (see Methods for further details).

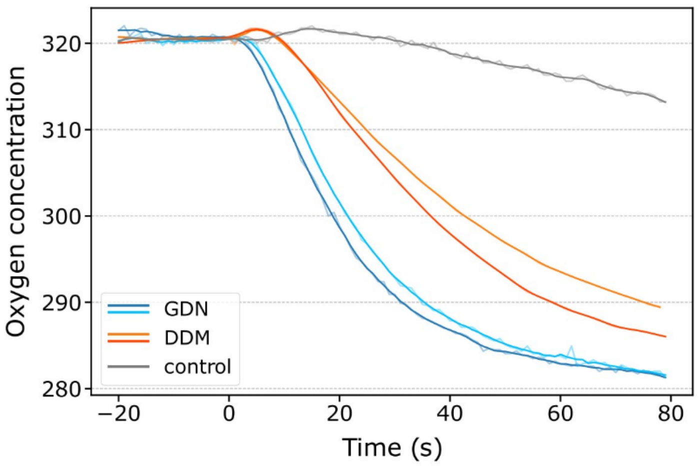

Figure S22. SC activity. Raw traces of the coupled activity measurements. The grey line shows the background O_2 reduction in the absence of SC and was subtracted from the other activities. Shaded lines represent the raw data, while solid lines show smoothed data.

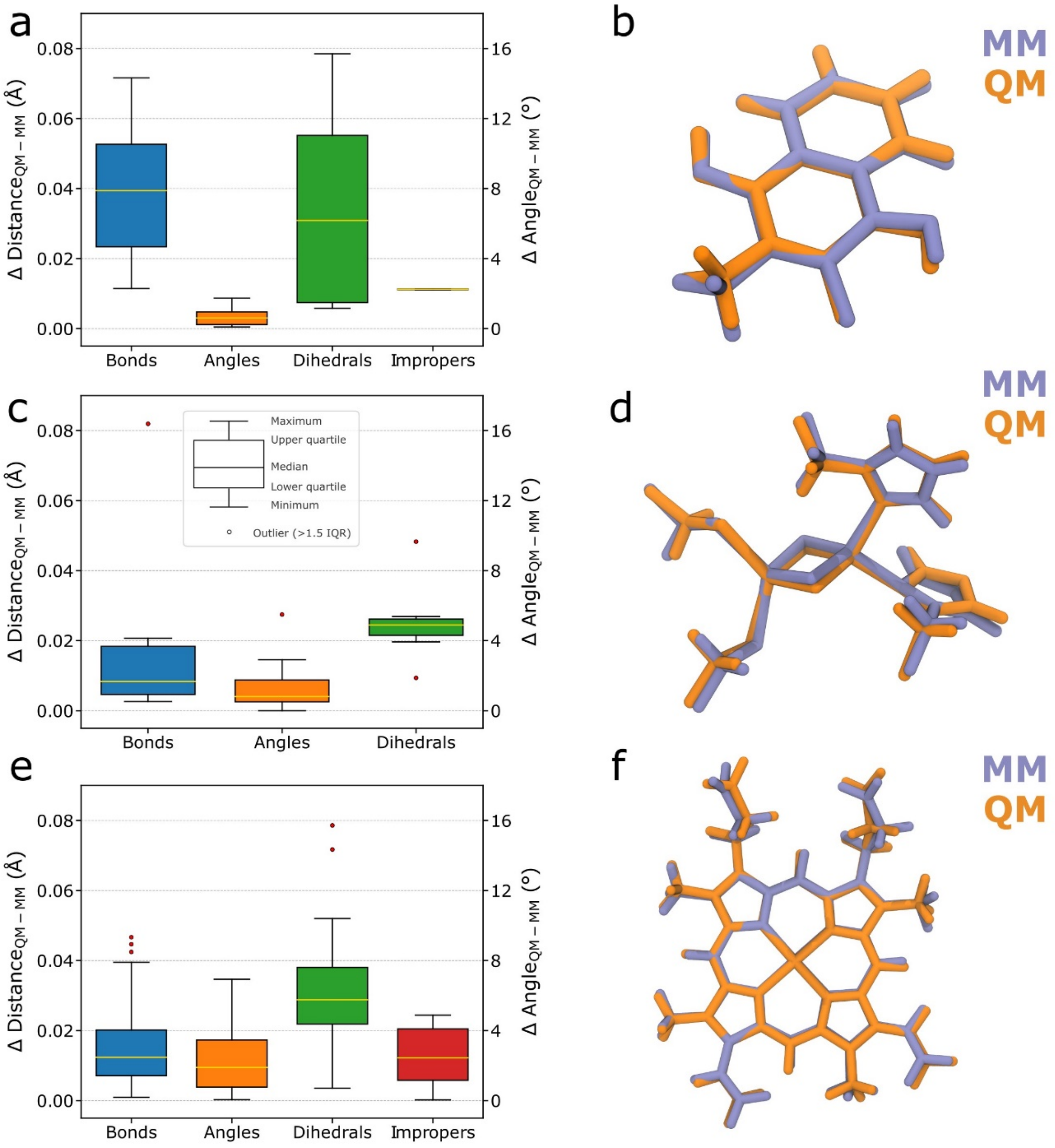

Figure S23. Force field validation. Comparison of differences in bond lengths and angles between MM (iceblue) and QM (orange) minimised structures of **a, b)** quinone, **c, d)** FeS centre, and **e, f)** heme *b*. See also Tables S12-S14. The MM and QM/MM optimisations were initiated from independent snapshots followed by a geometry optimisation. The MM system was minimised for 10000 steps, while the QM/MM system was minimised until energy convergence threshold of 0.006 kcal mol^{-1}, with the MM region restrained. The data raw data for the box plots can be found in Supplementary Tables 12-14.

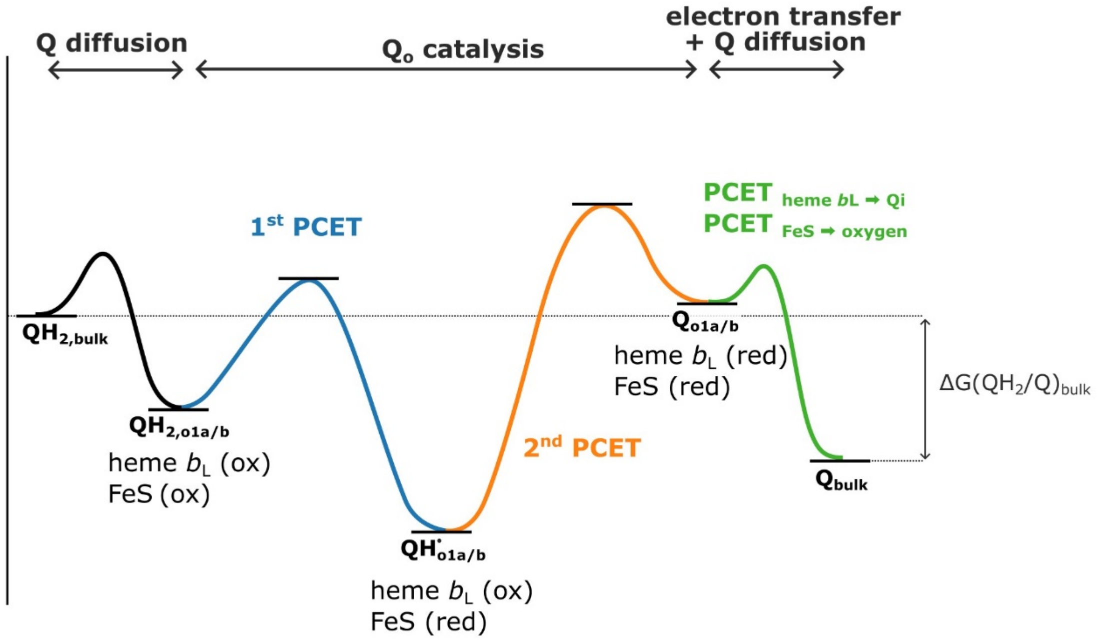

Figure S24. Schematic overview of charge transfer and quinone diffusion energetics. Representation of the energetics of the local PCET reaction at the Q_{1a} site, and the binding and release of the quinol/quinone from the membrane pool. Binding of quinol to the $QH_{2,o1b}$ and $QH_{2,o1a}$ sites from the membrane pool is exergonic by a few kcal mol^{-1} (based on K_m~120 µM (Ref.[2]) and calculated binding affinities (see also Supplementary Fig. 8). The two local binding sites could provide entropic stabilisation into the binding process. The QM/MM calculations predict that the initial PCET is weakly exergonic, followed by an endergonic PCET towards heme b_L. The green curve accounts for the subsequent electron transfer and proton translocation processes, as well as the quinone unbinding, including the cost for moving charges against the pmf (here 200 mV) based on experimental redox potentials[12]. The subsequent electron transfer and proton translocation processes are exergonic, whilst the whole process is driven by the reduction of O_2 at the BNC (E_m~820 mV).

Table S1. List of atomistic molecular dynamics simulations (*ca*. 10 μs). All simulations were based on the complete system, with around 900,000 atoms modelled in the symmetric (sym) and asymmetric (asym) states (see Methods). The oxidised FeS Rieske centre was modelled in a $Fe^{3+}Fe^{3+}$ state and deprotonated H355 (ε-tautomer), and the reduced state with $Fe^{3+}Fe^{2+}$ and both H335/H355 in their neutral form. Heme b_H, heme c_1, heme c_2, Cu_A, and the Cu of SOD-C were modelled in their oxidised states, whereas the binuclear centre was simulated in the P_M state ($Fe^{IV}=O^{2-}/Cu^{II}-OH^-/Tyr-O^•$). Simulations S17+S18 are performed of the canonical bovine C*c*O (*Bt* C*c*O, see Ref.[12]), and used as a reference for the D-channel hydration analysis (Fig. S10).

Simulation	System	Quinone	Q-site	FeS	heme b_L	Length (ns)	Protonation
**S1**	sym	QH_2	Q_{o1a}	ox	ox	500	$H110^0$ $D302^-$
**S1 replica**	sym	QH_2	Q_{o1a}	ox	ox	500	$H110^0$ $D302^-$
**S2**	sym	QH_2	Q_{o1a}	ox	ox	500	$H110^+$ $D302^-$
**S3**	sym	QH^-	Q_{o1a}	ox	ox	500	$H110^0$ $D302^-$
**S4**	sym	QH^-	Q_{o1a}	ox	ox	500	$H110^+$ $D302^-$
**S5**	sym	$QH^•$	Q_{o1a}	red	ox	500	$H110^0$ $D302^-$
**S6**	sym	$Q^{•/-}$	Q_{o1a}	red	ox	500	$H110^0$ $D302^0$
**S7**	sym	Q	Q_{o1a}	red	red	500	$H110^0$ $D302^0$
**S8**	sym	QH_2	Q_{o1a}	ox	ox	500	H110A – $D302^-$
**S9**	sym	QH_2	Q_{o1a}	ox	ox	500	H110A – $D302^0$
**S10**	sym	QH_2	Q_{o1a}	ox	ox	500	D309N ($H110^+$)
**S11**	sym	QH_2	Q_{o1a}	ox	ox	500	D302N ($H110^+$)
**S12**	sym	QH_2	Q_{o1a}	ox	ox	500	Y159F ($H110^+$ $D302^-$)
**S13**	asym	QH_2	Q_{o1a}	ox	ox	500	$H110^0$ $D302^-$
**S14**	asym	Q	Q_{o1a}	ox	ox	500	$H110^0$ $D302^-$
**S15**	sym	QH_2	Q_{o1b}	ox	ox	500	$H110^0$ $D302^-$
**S16**	sym	QH_2	Q_{o2}	ox	ox	500	$H110^0$ $D302^-$
**S17**	*Bt* C*c*O	-	-	-	-	500	see Ref. [1]
**S18**	*Bt* C*c*O	-	-	-	-	300	see Ref. [1]
					**Total:**	**9.3 μs**	

Table S2. List of subunits in the simulation models.

Subunit	Symmetric	Asymmetric
**Cox I**	yes	yes
**Cox II**	yes	yes
**Cox III**	yes	yes
**Cox IV**	yes	yes
**QcrA**	yes	yes
**QcrB**	yes	yes
**QcrC**	yes (closed+closed)	yes (open+closed)
**LpqE**	yes	no
**PRSAF1**	yes	yes
**AscF**	yes	yes
**AscG**	yes	yes
**SOD-C**	yes	yes

Table S3. List of residues modelled in non-standard protonation states based on Monte Carlo electrostatic calculations (see *Methods*). HSE: ε-protonated histidine; HSD: δ-protonated histidine; HSP: double protonated ($HisH^+$), charged histidine. All other histidine residues were modelled in the HSD state.

Simulation	Subunit	Residues
**S1,3,5,8,13-16**	QcrA	His335 (HSE), His355 (HSE)
	QcrC	Glu271
	Cox I	His264 (HSE), Glu266, Asp385
	Cox II	His232 (HSE), His281 (HSE)
**S6,7,9**	QcrA	His335 (HSE), His355 (HSE)
	QcrB	Asp302
	QcrC	Glu271
	Cox I	His264 (HSE), Glu266, Asp385
	Cox II	His232 (HSE), His281 (HSE)
**S2,4,10-12**	QcrA	His335 (HSE), His355 (HSE)
	QcrB	Asp302, His110 (HSP)
	QcrC	Glu271
	Cox I	His264 (HSE), Glu266, Asp385
	Cox II	His232 (HSE), His281 (HSE)

Table S4. Calculated electron transfer rates and estimated parameters. Rates in light grey were excluded from the kinetic model, since these reactions were modelled as irreversible to account for Q_i unbinding and oxygen reduction. A detailed version of the kinetic model was created to account for proton-coupled modulation of the BNC redox potential. [a] generic value, *cf.* Ref.[13]. [b] from Ref.[14] [c] Computed from the MD trajectories, [d] from Ref.[3]. PLS - unprotonated proton loading site (prior to proton uptake via the D-channel); PLSH - protonated proton-loading site (after proton uptake from the D-channel).

Reaction	Δ*G* (mV) E_m (mV)[b]	Reorganisation energy (eV)	Packing density (average)[c]	Forwards rate (s^{-1})	Backwards rate (s^{-1})
**QH_2 → FeS**	-234 (-74 → 160)	0.7[a]	0.74	$1.6 \cdot 10^{10}$	$1.9 \cdot 10^{7}$
**FeS → heme c_1**	60 (160 → 100)	0.7[a]	0.71	$2.0 \cdot 10^{3}$	$1.1 \cdot 10^{4}$
**heme c_1 → heme c_2**	0 (100 → 100)	0.7[a]	0.70	$1.5 \cdot 10^{6}$	$1.5 \cdot 10^{6}$
**heme c_2 → Cu_A**	-50 (100 → 150)	0.7[a]	0.71	$1.2 \cdot 10^{4}$	$3.0 \cdot 10^{3}$
**Cu_A → heme *a***	7 (150 → 143)	0.7[a]	0.71	$6.8 \cdot 10^{5}$	$6.8 \cdot 10^{5}$
**heme *a* → heme a_3**	-174 (143 → 317)	0.3[b]	0.69	$3.0 \cdot 10^{9}$	$2.4 \cdot 10^{7}$
**$QH^{\bullet}$ → heme b_L**	217 (-74 → -291)	0.7[a]	0.71	$8.5 \cdot 10^{4}$	$4.2 \cdot 10^{7}$
**heme b_L → heme b_H**	-128 (-291 → -163)	0.7[a]	0.66	$7.0 \cdot 10^{4}$	$1.8 \cdot 10^{3}$
**heme b_H → Q**	-89 (-163 → -74)	0.7[a]	0.71	$7.9 \cdot 10^{7}$	$6.2 \cdot 10^{6}$
**QH_2 (Q_{o1b}) → FeS**	-234 (-74 → 160)	0.7[a]	0.72	$2.3 \cdot 10^{6}$	$2.9 \cdot 10^{3}$
**$QH^{\bullet}$ (Q_{o1b}) → heme b_L**	217 (-74 → -291)	0.7[a]	0.72	$1.4 \cdot 10^{3}$	$6.9 \cdot 10^{5}$
**QH_2 (Q_{o2}) → FeS**	-234 (-74 → 160)	0.7[a]	0.72	$2.8 \cdot 10^{2}$	0.3
**$QH^{\bullet}$ (Q_{o2}) → heme b_L**	217 (-74 → -291)	0.7[a]	0.72	1.1	$5.3 \cdot 10^{2}$
**heme *a* → heme $a_3^{(PLS)}$**	340[d] (270 → -70)	0.3[b]	0.69	$2.6 \cdot 10^{5}$	$4.3 \cdot 10^{9}$
**heme *a* → heme $a_3^{(PLSH)}$**	-10[d] (270 → 280)	0.3[b]	0.69	$6.0 \cdot 10^{8}$	$4.5 \cdot 10^{8}$
**heme $a_3^{(PLSH)}$ → O_2**				$3.9 \cdot 10^{2}$ [d]	

Table S5. Calculated average redox midpoint potentials of cofactors based on MD simulations (see Table S1 for simulation number). All values are given in mV.

Simulation	Quinone	Rieske FeS	heme b_L	heme b_H
**WT$^+$ (S2)**	-150	127	-159	-43
**WT^0 (S1)**	-103	117	-149	-75
**H110A (S8)**	-115	101	-130	-86
**D309N (S10)**	-185	-2	-135	-75
**D302N (S11)**	-149	130	-146	-57
**Y159F (S12)**	-149	142	-150	-87

Table S6. CryoEM data collection, refinement, and validation statistics.

**Data collection, processing**	**Dataset 1**<br>PDB ID: 8OVD, EMBD: 17211	**Dataset 2**<br>PDB ID: 8OVC, EMBD: 17210
Voltage (kV)	300	300
Magnification	105000	105000
Electron exposure (e^- /Å^2)	48	48
Pixel size (Å)	0.828	0.846
Defocus range (μm)	-2.2 to -0.46	-2.2 to -0.4
Defocus step (μm)	-0.2	-0.2
Symmetry imposed	C2	None (C1)
Initial particle images (number)	808650	752 267
Final particle images (number)	208243	90 918
FSC threshold	0.143	0.143
Map resolution (Å)	2.3	2.8
**Refinement**		
CC (mask)	0.9	0.87
Resolution estimates (Å)		
d 99		
Masked	2.4	2.9
Unmasked	2.4	3.0
d FSC model, 0/0.143/0.5 (Å)		
Masked	2.2/2.3/2.4	2.7/2.8/3.0
Unmasked	2.3/2.3/2.6	2.8/2.8/3.1
Model composition		
Protein residues	5538	5611
Non-hydrogen atoms	47229	46720
Ligands	86	67
Water	614	249
B factors (Å^2)		
Min/max/mean		
Protein residues	2.23/55.34/16.55	7.08/167.03/27.64
Ligands	5.67/114.01/24.44	6.73/269.67/46.97
water	3.52/30.00/13.51	0.5/106.06/25.00
**Validation**		
MolProbity	1.67	1.78
Root mean square deviations		
Bond lengths (Å)	0.016	0.011
Bond angles (°)	1.225	1.041
Clash score	8	7.26
Poor rotamers (%)	0.14	0.13
Ramachandran plot		
Favoured (%)	96.9	94.4
Allowed (%)	3.03	5.47
Disallowed (%)	0.07	0.13

Table S7. Atom types and partial charges for MQ cofactors calculated in different oxidation states. QM models included one isoprenoid unit and were optimized at B3LYP/def2-SVP level of theory (see Methods) while quinone tail charges were taken from CHARMM36m force field[15].

	MQH2		MQ		MQH-		MQ		MQ-	
Name	**Type**	**q_i**	**Type**	**q_i**	**Type**	**q_i**	**Type**	**q_i**	**Type**	**q_i**
C1	CGD1	-0.045	CGD1	0.16	CGD1	-0.006	CGD1	0.064	CGD1	0.141965
C2	CGO5	0.08	CGO5	0.43	CGO5	0.15	CGO5	0.178	CGO5	0.275701
C3	CGR1	-0.045	CGR1	-0.1	CGR1	-0.074	CGR1	-0.026	CGR1	-0.06575
C4	CGR1	0.2	CGR1	-0.135	CGR1	-0.074	CGR1	-0.026	CGR1	-0.11067
C5	CGO5	-0.065	CGO5	0.6	CGO5	0.15	CGO5	0.178	CGO5	0.473501
C6	CGD1	0.075	CGD1	-0.35	CGD1	-0.006	CGD1	0.064	CGD1	-0.37547
C1M	CG31	-0.05	CG31	-0.35	CG31	-0.2315	CG31	-0.27	CG31	-0.34413
H1M	HGA3	0.035	HGA3	0.125	HGA3	0.05	HGA3	0.09	HGA3	0.097116
H2M	HGA3	0.035	HGA3	0.125	HGA3	0.05	HGA3	0.09	HGA3	0.097116
H3M	HGA3	0.035	HGA3	0.125	HGA3	0.05	HGA3	0.09	HGA3	0.097116
C7	CG21	-0.015	CG21	0.14	CG21	-0.1815	CG21	-0.18	CG21	0.166413
H71	HQA2	0.035	HQA2	0.055	HQA2	0.05	HQA2	0.09	HQA2	0.013277
H72	HQA2	0.035	HQA2	0.055	HQA2	0.05	HQA2	0.09	HQA2	0.013277
O2	OH1	-0.48	OGD3	-0.48	OH1	-0.554	OH1	-0.37	OGD3	-0.63018
H2	H	0.39	-	-	-	-	-	-	-	-
O5	OH1	-0.48	OGD3	-0.5	OH1	-0.525	OH1	-0.45	OGD3	-0.66222
H5	H	0.39	-	-	H	0.3	H	0.35	-	-
CB1	CGR1	-0.135	CGR1	-0.04	CGR1	-0.027	CGR1	-0.075	CGR1	-0.05829
HB1	HGR1	0.145	HGR1	0.11	HGR1	0.06	HGR1	0.11	HGR1	0.078915
CB2	CGR1	-0.2	CGR1	-0.135	CGR1	-0.232	CGR1	-0.155	CGR1	-0.18915
HB2	HGR1	0.165	HGR1	0.15	HGR1	0.1	HGR1	0.139	HGR1	0.132311
CB3	CGR1	-0.2	CGR1	-0.135	CGR1	-0.232	CGR1	-0.155	CGR1	-0.19027
HB3	HGR1	0.165	HGR1	0.15	HGR1	0.1	HGR1	0.139	HGR1	0.134768
CB4	CGR1	-0.135	CGR1	-0.04	CGR1	-0.027	CGR1	-0.075	CGR1	-0.07079
HB4	HGR1	0.145	HGR1	0.105	HGR1	0.06	HGR1	0.11	HGR1	0.081551
C8	CGD1	-0.325	CGD1	-0.325	CGD1	-0.15	CGD1	-0.15	CGD1	-0.19866
H81	HGA4	0.135	HGA4	0.14	HGA4	0.15	HGA4	0.15	HGA4	0.098895
C9	CEL1	0.115	CEL1	0.12	CEL1	-0.07	CEL1	-0.07	CEL1	-0.00635
C10	CTL3	-0.27	CTL3	-0.27	CTL3	-0.195	CTL3	-0.195	CTL3	-0.27
H101	HAL3	0.09	HAL3	0.09	HAL3	0.09	HAL3	0.09	HAL3	0.09
H102	HAL3	0.09	HAL3	0.09	HAL3	0.09	HAL3	0.09	HAL3	0.09
H103	HAL3	0.09	HAL3	0.09	HAL3	0.09	HAL3	0.09	HAL3	0.09
C11	CTL2	-0.11	CTL2	-0.105	CTL2	-0.11	CTL2	-0.11	CTL2	-0.105
H111	HAL2	0.09	HAL2	0.09	HAL2	0.09	HAL2	0.09	HAL2	0.09
H112	HAL2	0.09	HAL2	0.09	HAL2	0.09	HAL2	0.09	HAL2	0.09
C12	CTL2	-0.18	CTL2	-0.18	CTL2	-0.18	CTL2	-0.18	CTL2	-0.18
H121	HAL2	0.09	HAL2	0.09	HAL2	0.09	HAL2	0.09	HAL2	0.09
H122	HAL2	0.09	HAL2	0.09	HAL2	0.09	HAL2	0.09	HAL2	0.09
C13	CEL1	-0.15	CEL1	-0.15	CEL1	-0.15	CEL1	-0.15	CEL1	-0.15
H131	HEL1	0.15	HEL1	0.15	HEL1	0.15	HEL1	0.15	HEL1	0.15
C14	CEL1	-0.075	CEL1	-0.075	CEL1	-0.075	CEL1	-0.075	CEL1	-0.075
C15	CTL3	-0.27	CTL3	-0.27	CTL3	-0.27	CTL3	-0.27	CTL3	-0.27
H151	HAL3	0.09	HAL3	0.09	HAL3	0.09	HAL3	0.09	HAL3	0.09
H152	HAL3	0.09	HAL3	0.09	HAL3	0.09	HAL3	0.09	HAL3	0.09
H153	HAL3	0.09	HAL3	0.09	HAL3	0.09	HAL3	0.09	HAL3	0.09
C16	CTL2	-0.105	CTL2	-0.105	CTL2	-0.105	CTL2	-0.105	CTL2	-0.105
H161	HAL2	0.09	HAL2	0.09	HAL2	0.09	HAL2	0.09	HAL2	0.09
H162	HAL2	0.09	HAL2	0.09	HAL2	0.09	HAL2	0.09	HAL2	0.09
C17	CTL2	-0.18	CTL2	-0.18	CTL2	-0.18	CTL2	-0.18	CTL2	-0.18
H171	HAL2	0.09	HAL2	0.09	HAL2	0.09	HAL2	0.09	HAL2	0.09
H172	HAL2	0.09	HAL2	0.09	HAL2	0.09	HAL2	0.09	HAL2	0.09
C18	CEL1	-0.15	CEL1	-0.15	CEL1	-0.15	CEL1	-0.15	CEL1	-0.15
H181	HEL1	0.15	HEL1	0.15	HEL1	0.15	HEL1	0.15	HEL1	0.15
C19	CEL1	-0.075	CEL1	-0.075	CEL1	-0.075	CEL1	-0.075	CEL1	-0.075
C20	CTL3	-0.27	CTL3	-0.27	CTL3	-0.27	CTL3	-0.27	CTL3	-0.27
H201	HAL3	0.09	HAL3	0.09	HAL3	0.09	HAL3	0.09	HAL3	0.09
H202	HAL3	0.09	HAL3	0.09	HAL3	0.09	HAL3	0.09	HAL3	0.09
H203	HAL3	0.09	HAL3	0.09	HAL3	0.09	HAL3	0.09	HAL3	0.09
C21	CTL2	-0.105	CTL2	-0.105	CTL2	-0.105	CTL2	-0.105	CTL2	-0.105

H211	HAL2	0.09	HAL2	0.09	HAL2	0.09	HAL2	0.09	HAL2	0.09
H212	HAL2	0.09	HAL2	0.09	HAL2	0.09	HAL2	0.09	HAL2	0.09
C22	CTL2	-0.18	CTL2	-0.18	CTL2	-0.18	CTL2	-0.18	CTL2	-0.18
H221	HAL2	0.09	HAL2	0.09	HAL2	0.09	HAL2	0.09	HAL2	0.09
H222	HAL2	0.09	HAL2	0.09	HAL2	0.09	HAL2	0.09	HAL2	0.09
C23	CEL1	-0.15	CEL1	-0.15	CEL1	-0.15	CEL1	-0.15	CEL1	-0.15
H231	HEL1	0.15	HEL1	0.15	HEL1	0.15	HEL1	0.15	HEL1	0.15
C24	CEL1	-0.075	CEL1	-0.075	CEL1	-0.075	CEL1	-0.075	CEL1	-0.075
C25	CTL3	-0.27	CTL3	-0.27	CTL3	-0.27	CTL3	-0.27	CTL3	-0.27
H251	HAL3	0.09	HAL3	0.09	HAL3	0.09	HAL3	0.09	HAL3	0.09
H252	HAL3	0.09	HAL3	0.09	HAL3	0.09	HAL3	0.09	HAL3	0.09
H253	HAL3	0.09	HAL3	0.09	HAL3	0.09	HAL3	0.09	HAL3	0.09
C26	CTL2	-0.105	CTL2	-0.105	CTL2	-0.105	CTL2	-0.105	CTL2	-0.105
H261	HAL2	0.09	HAL2	0.09	HAL2	0.09	HAL2	0.09	HAL2	0.09
H262	HAL2	0.09	HAL2	0.09	HAL2	0.09	HAL2	0.09	HAL2	0.09
C27	CTL2	-0.18	CTL2	-0.18	CTL2	-0.18	CTL2	-0.18	CTL2	-0.18
H271	HAL2	0.09	HAL2	0.09	HAL2	0.09	HAL2	0.09	HAL2	0.09
H272	HAL2	0.09	HAL2	0.09	HAL2	0.09	HAL2	0.09	HAL2	0.09
C28	CEL1	-0.15	CEL1	-0.15	CEL1	-0.15	CEL1	-0.15	CEL1	-0.15
H281	HEL1	0.15	HEL1	0.15	HEL1	0.15	HEL1	0.15	HEL1	0.15
C29	CEL1	-0.075	CEL1	-0.075	CEL1	-0.075	CEL1	-0.075	CEL1	-0.075
C30	CTL3	-0.27	CTL3	-0.27	CTL3	-0.27	CTL3	-0.27	CTL3	-0.27
H301	HAL3	0.09	HAL3	0.09	HAL3	0.09	HAL3	0.09	HAL3	0.09
H302	HAL3	0.09	HAL3	0.09	HAL3	0.09	HAL3	0.09	HAL3	0.09
H303	HAL3	0.09	HAL3	0.09	HAL3	0.09	HAL3	0.09	HAL3	0.09
C31	CTL2	-0.105	CTL2	-0.105	CTL2	-0.105	CTL2	-0.105	CTL2	-0.105
H311	HAL2	0.09	HAL2	0.09	HAL2	0.09	HAL2	0.09	HAL2	0.09
H312	HAL2	0.09	HAL2	0.09	HAL2	0.09	HAL2	0.09	HAL2	0.09
C32	CTL2	-0.18	CTL2	-0.18	CTL2	-0.18	CTL2	-0.18	CTL2	-0.18
H321	HAL2	0.09	HAL2	0.09	HAL2	0.09	HAL2	0.09	HAL2	0.09
H322	HAL2	0.09	HAL2	0.09	HAL2	0.09	HAL2	0.09	HAL2	0.09
C33	CEL1	-0.15	CEL1	-0.15	CEL1	-0.15	CEL1	-0.15	CEL1	-0.15
H331	HEL1	0.15	HEL1	0.15	HEL1	0.15	HEL1	0.15	HEL1	0.15
C34	CEL1	-0.075	CEL1	-0.075	CEL1	-0.075	CEL1	-0.075	CEL1	-0.075
C35	CTL3	-0.27	CTL3	-0.27	CTL3	-0.27	CTL3	-0.27	CTL3	-0.27
H351	HAL3	0.09	HAL3	0.09	HAL3	0.09	HAL3	0.09	HAL3	0.09
H352	HAL3	0.09	HAL3	0.09	HAL3	0.09	HAL3	0.09	HAL3	0.09
H353	HAL3	0.09	HAL3	0.09	HAL3	0.09	HAL3	0.09	HAL3	0.09
C36	CTL2	-0.105	CTL2	-0.105	CTL2	-0.105	CTL2	-0.105	CTL2	-0.105
H361	HAL2	0.09	HAL2	0.09	HAL2	0.09	HAL2	0.09	HAL2	0.09
H362	HAL2	0.09	HAL2	0.09	HAL2	0.09	HAL2	0.09	HAL2	0.09
C37	CTL2	-0.18	CTL2	-0.18	CTL2	-0.18	CTL2	-0.18	CTL2	-0.18
H371	HAL2	0.09	HAL2	0.09	HAL2	0.09	HAL2	0.09	HAL2	0.09
H372	HAL2	0.09	HAL2	0.09	HAL2	0.09	HAL2	0.09	HAL2	0.09
C38	CEL1	-0.15	CEL1	-0.15	CEL1	-0.15	CEL1	-0.15	CEL1	-0.15
H381	HEL1	0.15	HEL1	0.15	HEL1	0.15	HEL1	0.15	HEL1	0.15
C39	CEL1	-0.075	CEL1	-0.075	CEL1	-0.075	CEL1	-0.075	CEL1	-0.075
C40	CTL3	-0.27	CTL3	-0.27	CTL3	-0.27	CTL3	-0.27	CTL3	-0.27
H401	HAL3	0.09	HAL3	0.09	HAL3	0.09	HAL3	0.09	HAL3	0.09
H402	HAL3	0.09	HAL3	0.09	HAL3	0.09	HAL3	0.09	HAL3	0.09
H403	HAL3	0.09	HAL3	0.09	HAL3	0.09	HAL3	0.09	HAL3	0.09
C41	CTL2	-0.105	CTL2	-0.105	CTL2	-0.105	CTL2	-0.105	CTL2	-0.105
H411	HAL2	0.09	HAL2	0.09	HAL2	0.09	HAL2	0.09	HAL2	0.09
H412	HAL2	0.09	HAL2	0.09	HAL2	0.09	HAL2	0.09	HAL2	0.09
C42	CTL2	-0.18	CTL2	-0.18	CTL2	-0.18	CTL2	-0.18	CTL2	-0.18
H421	HAL2	0.09	HAL2	0.09	HAL2	0.09	HAL2	0.09	HAL2	0.09
H422	HAL2	0.09	HAL2	0.09	HAL2	0.09	HAL2	0.09	HAL2	0.09
C43	CEL1	-0.15	CEL1	-0.15	CEL1	-0.15	CEL1	-0.15	CEL1	-0.15
H431	HEL1	0.15	HEL1	0.15	HEL1	0.15	HEL1	0.15	HEL1	0.15
C44	CEL1	-0.075	CEL1	-0.075	CEL1	-0.075	CEL1	-0.075	CEL1	-0.075
C45	CTL3	-0.27	CTL3	-0.27	CTL3	-0.27	CTL3	-0.27	CTL3	-0.27
H451	HAL3	0.09	HAL3	0.09	HAL3	0.09	HAL3	0.09	HAL3	0.09
H452	HAL3	0.09	HAL3	0.09	HAL3	0.09	HAL3	0.09	HAL3	0.09
H453	HAL3	0.09	HAL3	0.09	HAL3	0.09	HAL3	0.09	HAL3	0.09

C46	CTL2	-0.105	CTL2	-0.105	CTL2	-0.105	CTL2	-0.105	CTL2	-0.105
H461	HAL2	0.09	HAL2	0.09	HAL2	0.09	HAL2	0.09	HAL2	0.09
H462	HAL2	0.09	HAL2	0.09	HAL2	0.09	HAL2	0.09	HAL2	0.09
C47	CTL2	-0.18	CTL2	-0.18	CTL2	-0.18	CTL2	-0.18	CTL2	-0.18
H471	HAL2	0.09	HAL2	0.09	HAL2	0.09	HAL2	0.09	HAL2	0.09
H472	HAL2	0.09	HAL2	0.09	HAL2	0.09	HAL2	0.09	HAL2	0.09
C48	CEL1	-0.15	CEL1	-0.15	CEL1	-0.15	CEL1	-0.15	CEL1	-0.15
H481	HEL1	0.15	HEL1	0.15	HEL1	0.15	HEL1	0.15	HEL1	0.15
C49	CEL1	-0.075	CEL1	-0.075	CEL1	-0.075	CEL1	-0.075	CEL1	-0.075
C50	CTL3	-0.27	CTL3	-0.27	CTL3	-0.27	CTL3	-0.27	CTL3	-0.27
H501	HAL3	0.09	HAL3	0.09	HAL3	0.09	HAL3	0.09	HAL3	0.09
H502	HAL3	0.09	HAL3	0.09	HAL3	0.09	HAL3	0.09	HAL3	0.09
H503	HAL3	0.09	HAL3	0.09	HAL3	0.09	HAL3	0.09	HAL3	0.09
C51	CTL3	-0.27	CTL3	-0.27	CTL3	-0.27	CTL3	-0.27	CTL3	-0.27
H511	HAL3	0.09	HAL3	0.09	HAL3	0.09	HAL3	0.09	HAL3	0.09
H512	HAL3	0.09	HAL3	0.09	HAL3	0.09	HAL3	0.09	HAL3	0.09
H513	HAL3	0.09	HAL3	0.09	HAL3	0.09	HAL3	0.09	HAL3	0.09

Table S8. Atom types and partial charges for the FeS centre in different protonation and oxidation states relevant for the Rieske protein. Charges were calculated at B3LYP/def2-TZVP(Fe,S)/def2-SVP (C, H, N) using antiferromagnetic coupling (see Methods).

	**FeS (ox / His355 deprot.)**		**FeS (ox / His355 prot.)**		**FeS (red / His355 prot)**	
Name	**Type**	**q_i**	**Type**	**q_i**	**Type**	**q_i**
1FE1	FE	0.76679	FE	0.765737	FE	0.90225
1FE2	FE	0.705883	FE	0.480732	FE	0.561569
1S1	SX	-0.610877	SX	-0.523154	SX	-0.796441
1S2	SX	-0.587325	SX	-0.529569	SX	-0.79049
2SG	SG	-0.533667	SG	-0.581342	SG	-0.728898
2CB	CT2	0.157688	CT2	0.317065	CT2	0.353253
2HB1	HA2	-0.008193	HA2	-0.045394	HA2	-0.10796
2HB2	HA2	-0.031939	HA2	-0.060094	HA2	-0.093483
3SG	SG	-0.643177	SG	-0.580389	SG	-0.596307
3CB	CT2	0.344137	CT2	0.314246	CT2	0.117474
3HB1	HA2	-0.096595	HA2	-0.04341	HA2	-0.008411
3HB2	HA2	-0.066733	HA2	-0.060541	HA2	-0.006301
4ND1	NR21	-0.174536	NR21	-0.12125	NR21	-0.251098
4CE1	CPH2	0.062851	CPH2	-0.075513	CPH2	-0.028221
4HE1	HR1	0.063911	HR1	0.174195	HR1	0.140264
4NE2	NR1	-0.131438	NR1	-0.081999	NR1	-0.062767
4HE2	H	0.289456	H	0.300125	H	0.270709
4CD2	CPH1	-0.210706	CPH1	-0.174172	CPH1	-0.277703
4HD2	HR3	0.160563	HR3	0.177731	HR3	0.168261
4CG	CPH1	0.088007	CPH1	0.076366	CPH1	0.149183
4CB	CT2	-0.18	CT2	-0.18	CT2	-0.18
4HB1	HA2	0.09	HA2	0.09	HA2	0.09
4HB2	HA2	0.09	HA2	0.09	HA2	0.09
5ND1	NR21	-0.348993	NR21	-0.119708	NR21	-0.155227
5CE1	CPH2	0.262161	CPH2	-0.068861	CPH2	0.050428
5HE1	HR1	0.047032	HR1	0.161046	HR1	0.075988
5NE2	NR1	-0.587439	NR1	-0.069101	NR1	-0.134969
5HE2	-	-	H	0.298715	H	0.285119
5CD2	CPH1	0.234018	CPH1	-0.192088	CPH1	-0.193678
5HD2	HR3	0.007474	HR3	0.18145	HR3	0.145956
5CG	CPH1	-0.158354	CPH1	0.079178	CPH1	0.011501
5CB	CT2	-0.18	CT2	-0.18	CT2	-0.18
5HB1	HA2	0.09	HA2	0.09	HA2	0.09
5HB2	HA2	0.09	HA2	0.09	HA2	0.09

Table S9. Atom types and partial charges for heme *b* cofactor ligated to two His residues in reduced (Fe^{+2}) and oxidized state (Fe^{+3}) calculated at B3LYP/def2-TZVP(Fe)/def2-SVP(N,C,O,H) (See Methods).

	**heme *b* (red)**		**heme *b* (ox)**	
Name	**Type**	**q_i**	**Type**	**q_i**
1FE	FE	0.24	FE	0.48
1NA	NPH1	-0.18	NPH1	-0.094
1NB	NPH2	-0.18	NPH2	-0.094
1NC	NPH3	-0.18	NPH3	-0.094
1ND	NPH4	-0.18	NPH4	-0.094
1C1A	CPA	0.12	CPA	-0.038
1C2A	CPB	-0.06	CPB	0.048
1C3A	CPB	-0.06	CPB	0.11
1C4A	CPA	0.12	CPA	-0.051
1C1B	CPA	0.12	CPA	-0.038
1C2B	CPB	-0.06	CPB	0.048
1C3B	CPB	-0.06	CPB	0.11
1C4B	CPA	0.12	CPA	-0.051
1C1C	CPA	0.12	CPA	-0.038
1C2C	CPB	-0.06	CPB	0.048
1C3C	CPB	-0.06	CPB	0.11
1C4C	CPA	0.12	CPA	-0.051
1C1D	CPA	0.12	CPA	-0.038
1C2D	CPB	-0.06	CPB	0.048
1C3D	CPB	-0.06	CPB	0.11
1C4D	CPA	0.12	CPA	-0.051
1CHA	CPM	-0.1	CPM	-0.118
1HA	HA	0.1	HA	0.151
1CHB	CPM	-0.1	CPM	-0.118
1HB	HA	0.1	HA	0.151
1CHC	CPM	-0.1	CPM	-0.118
1HC	HA	0.1	HA	0.151
1CHD	CPM	-0.1	CPM	-0.118
1HD	HA	0.1	HA	0.151
1CMA	CT3	-0.27	CT3	-0.215
1HMA1	HA3	0.09	HA3	0.075
1HMA2	HA3	0.09	HA3	0.075
1HMA3	HA3	0.09	HA3	0.075
1CAA	CT2	-0.18	CT2	-0.351
1HAA1	HA2	0.09	HA2	0.175
1HAA2	HA2	0.09	HA2	0.175
1CBA	CT2	-0.28	CT2	-0.28
1HBA1	HA2	0.09	HA2	0.09
1HBA2	HA2	0.09	HA2	0.09
1CGA	CC	0.62	CC	0.62
1O1A	OC	-0.76	OC	-0.76
1O2A	OC	-0.76	OC	-0.76
1CMB	CT3	-0.27	CT3	-0.215
1HMB1	HA3	0.09	HA3	0.075
1HMB2	HA3	0.09	HA3	0.075
1HMB3	HA3	0.09	HA3	0.075
1CAB	CE1	-0.15	CE1	-0.166
1HAB	HE1	0.15	HE1	0.142
1CBB	CE2	-0.42	CE2	-0.325
1HBB1	HE2	0.21	HE2	0.169
1HBB2	HE2	0.21	HE2	0.169
1CMC	CT3	-0.27	CT3	-0.215
1HMC1	HA3	0.09	HA3	0.075
1HMC2	HA3	0.09	HA3	0.075
1HMC3	HA3	0.09	HA3	0.075
1CAC	CE1	-0.15	CE1	-0.166
1HAC	HE1	0.15	HE1	0.142
1CBC	CE2	-0.42	CE2	-0.325
1HBC1	HE2	0.21	HE2	0.169
1HBC2	HE2	0.21	HE2	0.169

1CMD	CT3	-0.27	CT3	-0.215
1HMD1	HA3	0.09	HA3	0.075
1HMD2	HA3	0.09	HA3	0.075
1HMD3	HA3	0.09	HA3	0.075
1CAD	CT2	-0.18	CT2	-0.351
1HAD1	HA2	0.09	HA2	0.175
1HAD2	HA2	0.09	HA2	0.175
1CBD	CT2	-0.28	CT2	-0.28
1HBD1	HA2	0.09	HA2	0.09
1HBD2	HA2	0.09	HA2	0.09
1CGD	CC	0.62	CC	0.62
1O1D	OC	-0.76	OC	-0.76
1O2D	OC	-0.76	OC	-0.76
2CB	CT2	-0.09	CT2	-0.09
2HB1	HA2	0.09	HA2	0.09
2HB2	HA2	0.09	HA2	0.09
2ND1	NR1	-0.36	NR1	-0.085
2HD1	H	0.32	H	0.378
2CG	CPH1	-0.05	CPH1	0.18
2CE1	CPH2	0.25	CPH2	0.183
2HE1	HR1	0.13	HR1	0.072
2NE2	NR2	-0.7	NR2	-0.448
2CD2	CPH1	0.22	CPH1	-0.358
2HD2	HR3	0.1	HR3	0.224
3CB	CT2	-0.09	CT2	-0.09
3HB1	HA2	0.09	HA2	0.09
3HB2	HA2	0.09	HA2	0.09
3ND1	NR1	-0.36	NR1	-0.085
3HD1	H	0.32	H	0.378
3CG	CPH1	-0.05	CPH1	0.18
3CE1	CPH2	0.25	CPH2	0.183
3HE1	HR1	0.13	HR1	0.072
3NE2	NR2	-0.7	NR2	-0.448
3CD2	CPH1	0.22	CPH1	-0.358
3HD2	HR3	0.1	HR3	0.224

Table S10. Atom types and partial charges for heme *c* cofactor covalently bond to two Cys and ligated to one His and one Met residue in reduced (Fe^{+2}) and oxidized state (Fe^{+3}) calculated at B3LYP/def2-TZVP(Fe)/def2-SVP(N,C,O,H) (See Methods).

		**heme *c* (Fe^{3+})**	**heme *c* (Fe^{2+})**
Name	**Type**	**q_i**	
1CB	CT2	-0.053097	-0.061453
1HB1	HA2	0.090000	0.090000
1HB2	HA2	0.090000	0.090000
1SG	S	-0.169121	-0.234224
2CB	CT2	-0.053097	-0.061453
2HB1	HA2	0.090000	0.090000
2HB2	HA2	0.090000	0.090000
2SG	S	-0.169121	-0.234224
3CB	CT2	-0.180000	-0.180000
3HB1	HA2	0.090000	0.090000
3HB2	HA2	0.090000	0.090000
3ND1	NR1	-0.417972	-0.295085
3HD1	H	0.380890	0.327175
3CG	CPH1	0.356471	0.142742
3CE1	CPH2	0.040851	-0.022940
3HE1	HR1	0.163413	0.162601
3NE2	NR2	-0.083064	-0.335722
3CD2	CPH1	-0.219132	-0.010508
3HD2	HR3	0.170850	0.163956
4CB	CT2	-0.097013	-0.049168
4HB1	HA2	0.050539	0.027309
4HB2	HA2	0.050539	0.027309
4CG	CT2	0.012302	0.042142
4HG1	HA2	0.067551	0.048487
4HG2	HA2	0.067551	0.048487
4SD	S	-0.026270	-0.230419
4CE	CT3	-0.352093	-0.293431
4HE1	HA3	0.162491	0.130283
4HE2	HA3	0.162491	0.130283
4HE3	HA3	0.162491	0.130283
5FE	FE	0.550532	0.578349
5NA	NPH	-0.015783	-0.046461
5NB	NPH	-0.015783	-0.046461
5NC	NPH	-0.015783	-0.046461
5ND	NPH	-0.015783	-0.046461
5C1A	CPA	-0.242566	-0.085379
5C2A	CPB	0.170125	0.032024
5C3A	CPB	0.106949	-0.029536
5C4A	CPA	-0.207860	0.003315
5C1B	CPA	-0.154400	-0.164522
5C2B	CPB	0.147977	0.090735
5C3B	CPB	0.091098	0.108366
5C4B	CPA	-0.379000	-0.230518
5C1C	CPA	-0.172063	-0.072131
5C2C	CPB	0.078355	-0.068566
5C3C	CPB	0.062196	0.106649
5C4C	CPA	-0.239994	-0.139557

5C1D	CPA	-0.153619	0.025869
5C2D	CPB	0.073582	-0.053398
5C3D	CPB	0.207932	0.083831
5C4D	CPA	-0.291582	-0.180732
5CHA	CPM	0.025075	-0.047706
5HA	HA	0.119250	0.082924
5CHB	CPM	0.025075	-0.047706
5HB	HA	0.119250	0.082924
5CHC	CPM	0.025075	-0.047706
5HC	HA	0.119250	0.082924
5CHD	CPM	0.025075	-0.047706
5HD	HA	0.119250	0.082924
5CMA	CT3	-0.270000	-0.270000
5HMA1	HA3	0.090000	0.090000
5HMA2	HA3	0.090000	0.090000
5HMA3	HA3	0.090000	0.090000
5CAA	CT2	-0.180000	-0.180000
5HAA1	HA2	0.090000	0.090000
5HAA2	HA2	0.090000	0.090000
5CMB	CT3	-0.270000	-0.270000
5HMB1	HA3	0.090000	0.090000
5HMB2	HA3	0.090000	0.090000
5HMB3	HA3	0.090000	0.090000
5CAB	CE1	0.019623	-0.092539
5HAB	HE1	0.115238	0.156411
5CBB	CE2	-0.180000	-0.180000
5HBB1	HE2	0.090000	0.090000
5HBB2	HE2	0.090000	0.090000
5CMC	CT3	-0.270000	-0.270000
5HMC1	HA3	0.090000	0.090000
5HMC2	HA3	0.090000	0.090000
5HMC3	HA3	0.090000	0.090000
5CAC	CE1	0.019623	-0.092539
5HAC	HE1	0.115238	0.156411
5CBC	CE2	-0.180000	-0.180000
5HBC1	HE2	0.090000	0.090000
5HBC2	HE2	0.090000	0.090000
5CMD	CT3	-0.270000	-0.270000
5HMD1	HA3	0.090000	0.090000
5HMD2	HA3	0.090000	0.090000
5HMD3	HA3	0.090000	0.090000
5CAD	CT2	-0.180000	-0.180000
5HAD1	HA2	0.090000	0.090000
5HAD2	HA2	0.090000	0.090000

Table S11. Atom types and partial charges for Cu active site in SodC ligated to three His residues and a hydroxyl for the oxidized state (Cu^{II}) obtained at B3LYP/def2-TZVP(Cu)/def2-SVP (N, C, O, H) level of theory (See Methods).

	**SOD-C - Cu**	
Name	**Type**	**q_i**
1CU	CUS	0.132217
5O1	OX	-0.451
5H1	HX	0.186886
2CB	CT2	-0.314446
2HB1	HA2	0.162662
2HB2	HA2	0.162662
2CG	CPH1	0.11583
2ND1	NX2	0.088193
2CE1	CPH2	0.112149
2HE1	HR1	0.099981
2NE2	NR1	-0.301441
2HE2	H	0.366666
2CD2	CPH1	-0.227735
2HD2	HR3	0.220606
3CB	CT2	-0.318703
3HB1	HA2	0.159357
3HB2	HA2	0.159357
3CG	CPH1	0.287385
3ND1	NR1	-0.336439
3HD1	H	0.366225
3CE1	CPH2	0.106954
3HE1	HR1	0.076508
3NE2	NX3	-0.011562
3CD2	CPH1	-0.375866
3HD2	HR3	0.213975
4CB	CT2	-0.353317
4HB1	HA2	0.169018
4HB2	HA2	0.169018
4CG	CPH1	0.350618
4ND1	NR1	-0.358268
4HD1	H	0.370906
4CE1	CPH2	0.012052
4HE1	HR1	0.172045
4NE2	NX4	-0.072554
4CD2	CPH1	-0.268605
4HD2	HR3	0.128666

Table S12. Comparison of MM and QM optimised quinone coordinates. Distances are given in Å and angles in degrees. See also Fig. S23 and Methods.

Type	**Atom 1**	**Atom 2**	**Atom 3**	**Atom 4**	**MM**	**QM**
Bond	C1	C2			1.567	1.496
Bond	C2	C3			1.537	1.514
Bond	C3	C4			1.478	1.423
Bond	C4	C5			1.568	1.511
Bond	C5	C6			1.558	1.509
Bond	C6	C1			1.417	1.365
Bond	C2	O2			1.336	1.349
Bond	C5	O5			1.374	1.363
Bond	C1	C1M			1.539	1.516
Bond	C3	CB1			1.471	1.417
Bond	CB1	CB2			1.426	1.401
Bond	CB2	CB3			1.422	1.394
Bond	CB3	CB4			1.412	1.396
Bond	CB4	C4			1.451	1.412
Bond	C6	C7			1.562	1.521
Angle	C1	C2	O2		122.7	121.0
Angle	C6	C5	O5		123.0	122.6
Angle	C1	C2	C3		122.0	121.1
Angle	CB1	CB2	CB3		120.1	120.2
Angle	C2	C3	CB1		125.1	124.0
Angle	C2	O2	H2		111.3	111.4
Angle	C5	O5	H5		107.4	106.8
Dihedral	CB1	CB2	CB3	CB4	2.2	1.0
Dihedral	C1	C2	C3	C4	-10.3	-11.7
Dihedral	C4	C5	C6	C7	163.7	165.7
Dihedral	C1	C2	O2	H2	177.7	166.5
Dihedral	C4	C5	O5	H5	169.3	185.0
Dihedral	C2	C3	C4	CB4	169.8	180.4
Improper	C2	C1	C3	O2	-2.8	-5.0
Improper	C5	C6	C4	O5	4.1	1.8

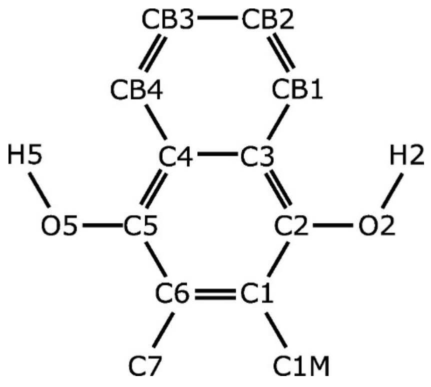

Table S13. Comparison of MM and QM optimised coordinates of the FeS Rieske centre. Distances are given in Å and angles in degrees. Leading numbers before the atom name denote the ligand (2/3 = Cys, 4/5 = His). See also Supplementary Fig. S23 and Methods.

Type	Atom 1	Atom 2	Atom 3	Atom 4	MM	QM
Bond	FE1	S1			2.224	2.227
Bond	FE1	S2			2.253	2.232
Bond	FE2	S1			2.226	2.222
Bond	FE2	S2			2.216	2.223
Bond	FE1	2SG			2.352	2.369
Bond	FE1	3SG			2.357	2.367
Bond	FE2	4ND1			2.053	2.135
Bond	FE2	5ND1			2.054	2.049
Angle	FE1	S1	FE2		73.5	73.8
Angle	FE1	S2	FE2		73.1	73.7
Angle	S1	FE1	S2		106.0	106.0
Angle	S1	FE2	S2		107.2	106.5
Angle	S1	FE1	2SG		107.9	107.3
Angle	S1	FE1	3SG		102.5	104.9
Angle	S2	FE1	2SG		102.2	104.6
Angle	S2	FE1	3SG		110.2	107.3
Angle	S1	FE2	4ND1		112.8	113.0
Angle	S1	FE2	5ND1		115.7	114.0
Angle	S2	FE2	4ND1		113.9	114.3
Angle	S2	FE2	5ND1		112.1	112.7
Angle	2SG	FE1	3-SG		126.6	125.4
Angle	FE1	2SG	2CB		100.8	99.9
Angle	FE1	3SG	3CB		101.8	100.4
Angle	FE2	4ND1	4CG		124.5	126.6
Angle	FE2	5ND1	5CG		137.9	132.4
Angle	FE2	4ND1	4CE1		123.5	124.1
Angle	FE2	5ND1	5CE1		114.5	114.9
Angle	4ND1	FE2	5ND1		95.1	96.4
Dihedral	FE1	S1	FE2	S2	4.1	-0.8
Dihedral	FE1	2SG	2CB	2HB1	-46.6	-56.3
Dihedral	FE1	3SG	3CB	3HB1	-71.7	-66.6
Dihedral	FE1	S1	FE2	4ND1	130.2	125.5
Dihedral	FE1	S1	FE2	5ND1	-121.8	-125.7

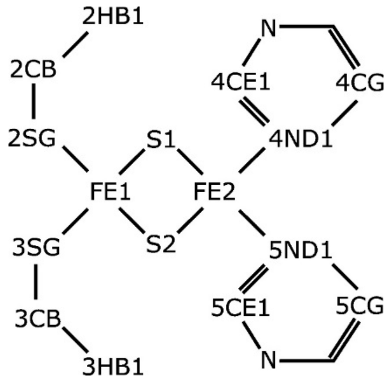

Table S14. Comparison of MM and QM optimised coordinates of heme *b*. Distances are given in Å and angles in degrees. See also Supplementary Fig. S23 and Methods.

Type	Atom 1	Atom 2	Atom 3	Atom 4	MM	QM
Bond	FE	NA			1.988	2.032
Bond	NA	C1A			1.378	1.376
Bond	C1A	C2A			1.446	1.464
Bond	C2A	C3A			1.360	1.378
Bond	C3A	C4A			1.439	1.456
Bond	C4A	NA			1.367	1.375
Bond	C4A	CHB			1.379	1.389
Bond	C3A	CMA			1.501	1.497
Bond	C2A	CAA			1.514	1.511
Bond	CAA	CBA			1.592	1.557
Bond	CBA	CGA			1.521	1.541
Bond	CGA	O1A			1.237	1.255
Bond	CGA	O2A			1.256	1.271
Bond	C1A	CHA			1.379	1.392
Bond	CHB	C1B			1.376	1397
Bond	C1B	C2B			1.453	1.443
Bond	C2B	C3B			1.385	1.383
Bond	C3B	C4B			1.474	1.452
Bond	FE	NB			2.003	2.011
Bond	C1B	NB			1.368	1.376
Bond	C4B	NB			1.376	1.373
Bond	C4B	CHC			1.389	1.390
Bond	C3B	CAB			1.422	1.461
Bond	CAB	CBB			1.356	1.345
Bond	C2B	CMB			1.526	1.492
Bond	FE	ND			1.985	1.996
Bond	ND	C4D			1.371	1.376
Bond	C4D	C3D			1.436	1.455
Bond	C3D	C2D			1.356	1.373
Bond	C2D	C1D			1.448	1.450
Bond	C1D	ND			1.365	1.379
Bond	C1D	CHD			1.369	1.388
Bond	C2D	CMD			1.520	1.501
Bond	C3D	CAD			1.491	1.495
Bond	CAD	CBD			1.565	1.534
Bond	CBD	CGD			1.487	1.533
Bond	CGD	O1D			1.230	1.250
Bond	CGD	O2D			1.258	1.267
Bond	C4D	CHA			1.382	1.395
Bond	CHD	C4C			1.380	1.391
Bond	C4C	C3C			1.481	1.461
Bond	C3C	C2C			1.384	1.383
Bond	C2C	C1C			1.456	1.446
Bond	FE	NC			1.993	2.027
Bond	C4C	NC			1.373	1.368
Bond	C1C	NC			1.367	1.374
Bond	C1C	CHC			1.380	1.390
Bond	C3C	CAC			1.423	1.458
Bond	CHC	CBC			1.350	1.346
Bond	C2C	CNC			1.535	1.493
Angle	NA	FE	NB		90.2	90.0
Angle	FE	NA	C4A		127.5	126.7
Angle	NA	C4A	C3A		112.1	110.7
Angle	NA	C4A	CHB		122.9	125.1
Angle	C4A	C3A	C2A		106.6	106.7
Angle	C4A	CHB	C1B		129.0	125.6
Angle	C4A	C3A	CMA		125.1	124.5
Angle	C3A	C2A	CAA		128.7	126.7
Angle	C2A	CAA	CBA		112.5	114.9
Angle	CAA	CBA	CGA		119.5	113.3

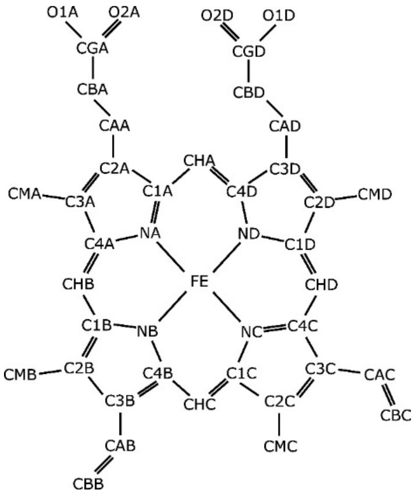

Angle	CBA	CGA	O1A		124.4	119.9
Angle	O1A	CGA	O2A		116.0	122.5
Angle	FE	NB	C1B		127.8	127.2
Angle	NB	C4B	C3B		112.7	110.1
Angle	NB	C4B	CHC		122.4	125.4
Angle	C1B	C2B	CMB		124.7	127.1
Angle	C2B	C3B	CAB		131.8	125.1
Angle	C3B	CAB	CBB		129.9	128.2
Angle	ND	FE	NC		90.7	89.5
Angle	FE	ND	C1D		127.4	127.2
Angle	ND	C1D	C2D		112.8	110.6
Angle	ND	C1D	CHD		122.0	125.6
Angle	C1D	C2D	C3D		104.6	106.4
Angle	C1D	CHD	C4C		130.6	125.0
Angle	C1D	C2D	CMD		127.2	126.3
Angle	C2D	C3D	CAD		127.7	127.8
Angle	C3D	CAD	CBD		113.3	112.0
Angle	CAD	CBD	CGD		113.9	115.7
Angle	CBD	CGD	O1D		116.7	116.6
Angle	O1D	CGD	O2D		118.1	125.1
Angle	FE	NC	C4C		127.4	127.7
Angle	NC	C1C	C2C		112.2	110.7
Angle	NC	C1C	CHC		122.4	125.1
Angle	C1C	C2C	CMC		123.6	126.1
Angle	C4C	C3C	CAC		130.1	124.3
Angle	C3C	CAC	CBC		128.2	128.4
Dihedral	NB	FE	NA	C1A	182.9	178.7
Dihedral	FE	NA	C4A	C3A	179.1	184.9
Dihedral	NA	C4A	CHB	C1B	8.3	-0.3
Dihedral	NA	C4A	C3A	C2A	3.5	0.5
Dihedral	NA	C4A	C3A	CMA	-176.2	-179.9
Dihedral	FE	NA	C1A	CHA	-1.7	-7.3
Dihedral	NA	FE	NB	C4B	-177.5	-170.3
Dihedral	FE	NB	C1B	C2B	177.3	187.7
Dihedral	NB	C1B	CHB	C4A	-4.4	-5.1
Dihedral	NB	C1B	C2B	C3B	0.6	2.0
Dihedral	NB	C1B	C2B	CMB	184.8	178.7
Dihedral	FE	NB	C4B	CHC	1.7	-14.0
Dihedral	NC	FE	ND	C4D	-178.5	-172.7
Dihedral	FE	ND	C1D	C2D	-173.1	-177.9
Dihedral	ND	C1D	CHD	C4C	-0.8	-5.5
Dihedral	ND	C1D	C2D	C3D	-2.0	2.3
Dihedral	ND	C1D	C2D	CMD	177.3	181.7
Dihedral	FE	ND	C4D	CHA	-2.9	-10.0
Dihedral	ND	FE	NC	C1C	182.5	176.0
Dihedral	FE	NC	C4C	C3C	193.7	179.4
Dihedral	NC	C4C	CHB	C1D	158.9	168.8
Dihedral	NC	C4C	C3C	C2C	-3.1	2.8
Dihedral	NC	C4C	C3C	CAC	175.0	184.4
Dihedral	FE	NC	C1C	CHC	-7.5	-2.8
Improper	C2A	C1A	C3A	CAA	0.7	1.1
Improper	C3A	C2A	C4A	CMA	-0.2	0.2
Improper	NA	C1A	C4A	FE	-0.9	-2.1
Improper	C4A	C3A	CHB	NA	-3.1	0.9
Improper	C1A	NA	C2A	CHA	-0.4	-0.6
Improper	C3B	C4B	C2B	CAB	-1.4	1.2
Improper	C2B	C3B	C1B	CMB	-2.2	1.7
Improper	NB	C4B	C1B	FE	0.2	-4.2
Improper	C4B	NB	C3B	CHC	-1.1	-2.2
Improper	C3D	C4D	C2D	CAD	2.0	-2.2
Improper	C2D	C3D	C1D	CMD	0.4	0.3
Improper	ND	C4D	C1D	FE	-2.1	-1.0
Improper	C1D	C2D	CHD	ND	0.2	-2.3
Improper	C4D	ND	C3D	CHA	2.1	-2.7

Improper	C2C	C1C	C3C	CMC	2.8	0.3
Improper	C3C	C2C	C4C	CAC	0.9	-0.9
Improper	NC	C1C	C4C	FE	-4.5	-0.3
Improper	C1C	NC	C2C	CHC	3.6	-0.7

Table S15. Calculated pK_a values of selected residues in the Q_{o1a} and Q_i sites. The calculations were performed for Complex III (PDB ID: 6ADQ)[2] with bound quinone before (6adq) and after (6adq opt) geometry optimisation of sidechains. The table reports values for each protomer.

Residue	**6adq**	**6adq opt**
Lys32	9.5/9.4	2.7/2.8
Glu44	9.7/9.7	1.5/5
His110	>14/>14	<0/<0
Lys260	1.7/1.8	3.5/3.4
Asp302	0.0/0.0	<0/<0
Asp309	3.6/3.9	2.3/2.0

SI References

1. Wiseman, B. *et al.* Structure of a functional obligate complex III_2IV_2 respiratory supercomplex from Mycobacterium smegmatis. *Nat. Struct. Mol. Biol.* **25**, 1128-1136 (2018).
2. Gong, H. *et al.* An electron transfer path connects subunits of a mycobacterial respiratory supercomplex. *Science* **362**, eaat8923 (2018).
3. Spagnolo, L. *et al.* Unique Features of the sodC-encoded Superoxide Dismutase from Mycobacterium tuberculosis, a Fully Functional Copper-containing Enzyme Lacking Zinc in the Active Site. *J. Biol. Chem.* **279**, 33447-33455 (2004).
4. Lange, C., Nett, J.H., Trumpower, B.L. & Hunte, C. Specific roles of protein-phospholipid interactions in the yeast cytochrome bc_1 complex structure. *EMBO J.* **20**, 6591-600 (2001).
5. Letts, J.A. *et al.* Structures of Respiratory Supercomplex I+III2 Reveal Functional and Conformational Crosstalk. *Mol Cell* **75**, 1131-1146 e6 (2019).
6. Huang, L.-S., Cobessi, D., Tung, E.Y. & Berry, E.A. Binding of the Respiratory Chain Inhibitor Antimycin to the Mitochondrial bc_1 Complex: A New Crystal Structure Reveals an Altered Intramolecular Hydrogen-bonding Pattern. *J. Mol. Biol.* **351**, 573-597 (2005).
7. Yanofsky, D.J. *et al.* Structure of mycobacterial $CIII_2CIV_2$ respiratory supercomplex bound to the tuberculosis drug candidate telacebec (Q203). *eLife* **10**, e71959. (2021).
8. Zhou, S. *et al.* Structure of Mycobacterium tuberculosis cytochrome *bcc* in complex with Q203 and TB47, two anti-TB drug candidates. *eLife* **10**(2021).
9. Guo, R. *et al.* Architecture of Human Mitochondrial Respiratory Megacomplex $I_2III_2IV_2$. *Cell* **170**, 1247-1257.e12 (2017).
10. Chovancova, E. *et al.* CAVER 3.0: A Tool for the Analysis of Transport Pathways in Dynamic Protein Structures. *PLoS Comput. Biol.* **8**, e1002708 (2012).
11. Sievers, F. *et al.* Fast, scalable generation of high-quality protein multiple sequence alignments using Clustal Omega. *Mol. Syst. Biol.* **7**, 539 (2011).
12. Di Trani, J.M. *et al.* Structural basis of mammalian complex IV inhibition by steroids. *Proc. Nat. Acad. Sci. U S A* **119**, e2205228119 (2022).
13. Moser, C.C., Page, C.C. & Dutton, P.L. Darwin at the molecular scale: selection and variance in electron tunnelling proteins including cytochrome *c* oxidase. *Phil. Trans. R. Soc. B* **361**, 1295-1305 (2006).
14. Kaila, V.R.I., Johansson, M.P., Sundholm, D. & Wikström, M. Interheme electron tunneling in cytochrome c oxidase. *Proc. Nat. Acad. Sci. USA* **107**, 21470-21475 (2010).
15. Huang, J. *et al.* CHARMM36m: an improved force field for folded and intrinsically disordered proteins. *Nat. Methods* **14**, 71-73 (2017).
