# Supplementary material for: Long-range charge transfer mechanism of the III2IV2 mycobacterial supercomplex
Source: Nat Commun. 2024 Jun 20;15:5276. doi: 10.1038/s41467-024-49628-9 (PMC11189923; doi:10.1038/s41467-024-49628-9)
Supplement: Supplementary file 3 — Description of Additional Supplementary Files [file 41467_2024_49628_MOESM3_ESM.pdf]

Description of Additional Supplementary Files

File Name: Supplementary Movie 1

Description: Molecular dynamics of the SC from M. smegmatis.

File Name: Supplementary Movie 2

Description: Quinol oxidation-driven PCET to the FeS centre based on QM/MM free energy calculations.

File Name: Supplementary Movie 3

Description: Quinol oxidation-driven PCET to the heme bL based on QM/MM free energy calculations.

File Name: Supplementary Movie 4

Description: Protonation dynamics of the fully reduced Qi based on QM/MM MD simulations.

File Name: Supplementary Movie 5

Description: Protonation dynamics of the semiquinone Qi based on QM/MM MD simulations.
