# Supplementary material for: Long-range charge transfer mechanism of the III2IV2 mycobacterial supercomplex
Source: Nat Commun. 2024 Jun 20;15:5276. doi: 10.1038/s41467-024-49628-9 (PMC11189923; doi:10.1038/s41467-024-49628-9)
Supplement: Supplementary file 9 — Reporting Summary [file 41467_2024_49628_MOESM9_ESM.pdf]

nature portfolio

Corresponding author(s): Ville Kaila

Last updated by author(s): Apr 17, 2024

Reporting Summary

Nature Portfolio wishes to improve the reproducibility of the work that we publish. This form provides structure for consistency and transparency in reporting. For further information on Nature Portfolio policies, see our Editorial Policies and the Editorial Policy Checklist.

Statistics

For all statistical analyses, confirm that the following items are present in the figure legend, table legend, main text, or Methods section.

n/a	Confirmed	
☐	☒	The exact sample size (*n*) for each experimental group/condition, given as a discrete number and unit of measurement
☐	☒	A statement on whether measurements were taken from distinct samples or whether the same sample was measured repeatedly
☒	☐	The statistical test(s) used AND whether they are one- or two-sided<br>*Only common tests should be described solely by name; describe more complex techniques in the Methods section.*
☒	☐	A description of all covariates tested
☒	☐	A description of any assumptions or corrections, such as tests of normality and adjustment for multiple comparisons
☐	☒	A full description of the statistical parameters including central tendency (e.g. means) or other basic estimates (e.g. regression coefficient) AND variation (e.g. standard deviation) or associated estimates of uncertainty (e.g. confidence intervals)
☒	☐	For null hypothesis testing, the test statistic (e.g. *F*, *t*, *r*) with confidence intervals, effect sizes, degrees of freedom and *P* value noted<br>*Give P values as exact values whenever suitable.*
☒	☐	For Bayesian analysis, information on the choice of priors and Markov chain Monte Carlo settings
☒	☐	For hierarchical and complex designs, identification of the appropriate level for tests and full reporting of outcomes
☒	☐	Estimates of effect sizes (e.g. Cohen's *d*, Pearson's *r*), indicating how they were calculated

Our web collection on statistics for biologists contains articles on many of the points above.

Software and code

Policy information about availability of computer code

Data collection	Smart EPU v2.12.1
Data analysis	Cryosparc v3, Cryosparc v4, CTFFIND4, PHENIX 1.18.2, Coot 0.8.9.2, Refmac 5.8.0257, MolProbity, VMD 1.93-1.94, UCSF ChimeraX 1.5, NAMD2.14, NAMD3 alpha9, CHARMM c38b , SWISS-MODEL, TURBOMOLE v.7.5, Karlsberg+, APBS 1.5, Clustal Omega, Uniprot, MDAnalysis 2.0.0, COPASI 4.35, WATCLUST v0.1, Jalview 2.11, CAVER3, PDB database (PDB IDs: 6HWH, 6ADQ, 7RH5, 7RH6, 7RH7, 7E1V, 7QHO, 7COH, 7ATE, 7AU6, 6Q9E, 1PP9, 1KYO, 1PZS), EMDB (IDs: 0289, 9610)

For manuscripts utilizing custom algorithms or software that are central to the research but not yet described in published literature, software must be made available to editors and reviewers. We strongly encourage code deposition in a community repository (e.g. GitHub). See the Nature Portfolio guidelines for submitting code & software for further information.

Data

Policy information about availability of data

All manuscripts must include a data availability statement. This statement should provide the following information, where applicable:

- Accession codes, unique identifiers, or web links for publicly available datasets
- A description of any restrictions on data availability
- For clinical datasets or third party data, please ensure that the statement adheres to our policy

Provide your data availability statement here.

Research involving human participants, their data, or biological material

Policy information about studies with human participants or human data. See also policy information about sex, gender (identity/presentation), and sexual orientation and race, ethnicity and racism.

Reporting on sex and gender	Not applicable
Reporting on race, ethnicity, or other socially relevant groupings	Not applicable
Population characteristics	Not applicable
Recruitment	Not applicable
Ethics oversight	Not applicable

Note that full information on the approval of the study protocol must also be provided in the manuscript.

Field-specific reporting

Please select the one below that is the best fit for your research. If you are not sure, read the appropriate sections before making your selection.

☒ Life sciences ☐ Behavioural & social sciences ☐ Ecological, evolutionary & environmental sciences

For a reference copy of the document with all sections, see nature.com/documents/nr-reporting-summary-flat.pdf

Life sciences study design

All studies must disclose on these points even when the disclosure is negative.

Sample size	Sample sizes were not predetermined. Cryo-EM particles were collected until convergence of resolution and structural properties. MD simulations were run long enough to ensure convergence of properties like hydration, etc.
Data exclusions	Cryo-EM particles belonging to classes with "broken" protein were removed from the structural analysis. No simulation data was excluded.
Replication	Each cryo-EM map was generated based on one data set, with both dataset showing similar results. Due to the large systems size and amount of studied states, MD simulations were run without replicas, but the C2 symmetry of the system provides an implicit replica. Analysis of MD properties were carried out on 1000 evenly spaced snapshots, with both protomers of the enzymes giving similar results. Quantum chemical simulations were carried out on a single, selected snapshot from MD simulations, due to the high computational cost. Activity measurements in different detergent were performed with at least 4 technical repeats.
Randomization	Not relevant. Structural biology or computational simulations do not use experimental groups.
Blinding	Not relevant. Structural biology or computational simulations do not use experimental groups.

Reporting for specific materials, systems and methods

We require information from authors about some types of materials, experimental systems and methods used in many studies. Here, indicate whether each material, system or method listed is relevant to your study. If you are not sure if a list item applies to your research, read the appropriate section before selecting a response.

Materials & experimental systems

n/a	Involved in the study	
☒	☐	Antibodies
☒	☐	Eukaryotic cell lines
☒	☐	Palaeontology and archaeology
☒	☐	Animals and other organisms
☒	☐	Clinical data
☒	☐	Dual use research of concern
☒	☐	Plants

Methods

n/a	Involved in the study	
☒	☐	ChIP-seq
☒	☐	Flow cytometry
☒	☐	MRI-based neuroimaging

Plants

Seed stocks	Not applicable
Novel plant genotypes	Not applicable
Authentication	Not applicable
